# Integrating curation into scientific publishing to train AI models

**DOI:** 10.1093/bioinformatics/btaf685

**Published:** 2025-12-27

**Authors:** Jorge Abreu-Vicente, Hannah Sonntag, Thomas Eidens, Cassie S Mitchell, Thomas Lemberger

**Affiliations:** EMBO, Heidelberg 69117, Germany; EMBO, Heidelberg 69117, Germany; EMBO, Heidelberg 69117, Germany; Department of Biomedical Engineering, Georgia Institute of Technology and Emory University School of Medicine, Atlanta, GA 30332, United States; EMBO, Heidelberg 69117, Germany

## Abstract

**Motivation:**

High-throughput extraction and structured labeling of data from academic articles are crucial for enabling downstream machine learning applications and secondary analyses. Current approaches lack integration with the publishing process and comprehensive annotation of experimental roles and methodologies alongside bioentity recognition.

**Results:**

We embedded multimodal data curation into the academic publishing process to annotate segmented figure panels and captions, combining natural language processing with authors’ feedback to increase annotation accuracy. The resulting dataset, SourceData-NLP, comprises over 620 000 annotated biomedical entities, curated from 18 689 figures in 3223 articles in molecular and cell biology. Annotations include eight classes of bioentities (small molecules, gene products, subcellular components, cell lines, cell types, tissues, organisms, and diseases), plus additional classes that delineate the entities’ roles in experimental designs and methodologies. We evaluate the utility of the dataset for training AI models using named-entity recognition, segmentation of figure captions into their constituent panels, and a novel context-dependent semantic task that assesses whether an entity is a controlled intervention target or a measurement object. We also demonstrate multi-modal applications for segmenting figures into panel images and their corresponding captions.

**Availability and implementation:**

Trained models are available at https://huggingface.co/EMBO. The SourceData-NLP dataset and code are available at https://github.com/source-data/soda-data, https://github.com/source-data/soda-model, and https://github.com/source-data/soda_image_segmentation.

## 1 Introduction

Scientific productivity in the life sciences has reached unprecedented levels. PubMed alone has over 38 million articles. Furthermore, millions of new articles are indexed every year in PubMed and posted on an expanding range of preprint platforms. This productivity poses challenges for researchers who must remain current with breakthroughs not only in their own disciplines but also in the broader scientific ecosystem (Lu 2011). The so-called “curse of specialization”, in which researchers become increasingly siloed in narrow domains, further hinders interdisciplinary insight and collaboration (Hall *et al.* 2008, Marrone and Linnenluecke 2020).

To address these challenges, the integration of scientific knowledge across the literature into usable structured and queryable resources is an important objective in biomedical research (Smith *et al.* 2007, Wilkinson *et al.* 2016, Liechti et al. 2017, Brown *et al.* 2019). To this end, natural language processing (NLP) plays a crucial role as it enables the large-scale computational processing of the literature to extract structured information from unstructured text. Classical tasks include named-entity recognition (NER) and named-entity linking (NEL). NER focuses on identifying entities of interest (e.g. genes, proteins, chemicals) in text, whereas NEL disambiguates these entities and assigns them standardized terms from controlled vocabularies or database identifiers (Bikel *et al.* 1997, Hoffart *et al.* 2011). These processes can potentially improve the reliability and rigor of reporting scientific findings by resolving potential ambiguities in terminologies or unclear concepts, which are frequent issues in the biomedical domain (Schuemie *et al.* 2005, Jonquet *et al.* 2009). In turn, by supporting better organization, retrieval and integration of published information, it can alleviate information overload while fostering reproducibility and transparency (Neves and Leser 2014).

The field of biocuration has made significant efforts to compile large, annotated datasets (e.g. Perera *et al.* 2020, Zhao *et al.* 2020, Fries *et al.* 2022), yet these resources remain relatively small and specialized, often derived from abstracts. In the life sciences, figures provide a crucial, albeit not exclusive, source of evidence that supports the claims of a paper, with captions providing detailed, natural-language descriptions of the respective experiments. While tables and supplementary materials are important additional sources of evidence, the present work focuses on the main figures of a paper (Li *et al.* 2021). This offers an opportunity to use figure curation to capture important information about experimental design and research data in a more granular way than abstracts allow.

Embedding curation within the publication process, including the involvement of authors, can be challenging (Wei *et al.* 2012, Horbach 2020), as it introduces complexities in the publishing workflow, causing possible delays and additional work for authors and editorial staff. However, it also offers the opportunity to resolve terminological ambiguities at the source (Li *et al.* 2016) and to make curated data publicly available immediately upon publication, thus facilitating information retrieval and secondary analyses. Furthermore, the publication of curated structured data provides a source for labeled data useful for training or benchmarking models in artificial intelligence.

Here, we present a large multimodal dataset, SourceData-NLP, built by integrating the curation of scientific figures directly into the publication workflow. The dataset couples each figure panel image with its corresponding caption segment, annotated according to the SourceData curation framework (Liechti et al. 2017) and refined with feedback from the authors. These annotations span biological entities from small molecules to tissues and entire organisms, making the dataset inherently multiscale. A distinguishing feature of SourceData-NLP is its delineation of entities that are measured versus those that are the target of a controlled experimental intervention, thereby capturing key design aspects of perturbation experiments for causal testing. This level of detail enables deeper insight into the nature of the reported findings, setting SourceData-NLP apart from other biomedical corpora.

The resulting dataset, SourceData-NLP, provides an integrated, searchable, and ready-to-use database for secondary analyses and tool development. To highlight the utility of SourceData-NLP, we conduct an analysis of the performance of the two best-performing transformer-based (Vaswani *et al.* 2017) models for biomedical NER (BioLinkBERT, Yasunaga *et al.* 2022, PubmedBERT, Gu *et al.* 2022). Additionally, we demonstrate the utility of our dataset to build object detection-based figure segmentation models that separate scientific figure images into their constituent panels and match them to the corresponding parts of the figure caption. To facilitate access for researchers to SourceData-NLP, we openly release the raw data in XML and graph database (neo4j) formats, machine-learning pre-tokenized data, the trained models, and the figure segmentation data and models.

In summary, SourceData-NLP provides a unique multimodal resource for biomedical text mining and a blueprint for embedding data curation into the publication lifecycle. By capturing experimental context at the point of publication and pairing figures with structured descriptive metadata, this approach should greatly facilitate data discovery, reproducibility, and integrative research in biomedical science.

## 2 Materials and methods

We include the materials and methods section as an online supplementary material. Please see Supplementary Material A, available as supplementary data at *Bioinformatics* online.

## 3 Results

In this section, we provide a comprehensive overview of the generation of the SourceData-NLP dataset, emphasizing its key characteristics. We then discuss the outcomes of our analyses to highlight the utility of SourceData-NLP. We first present the results for NER benchmarking. We then introduce a novel semantic task aimed at interpreting the experimental roles of bioentities within experimental setups. Finally, we extend our analysis to include scientific figure segmentation and panel-caption text matching, using a combination of object detection and generative AI models.

### 3.1 Dataset generation

**Figure 1.**
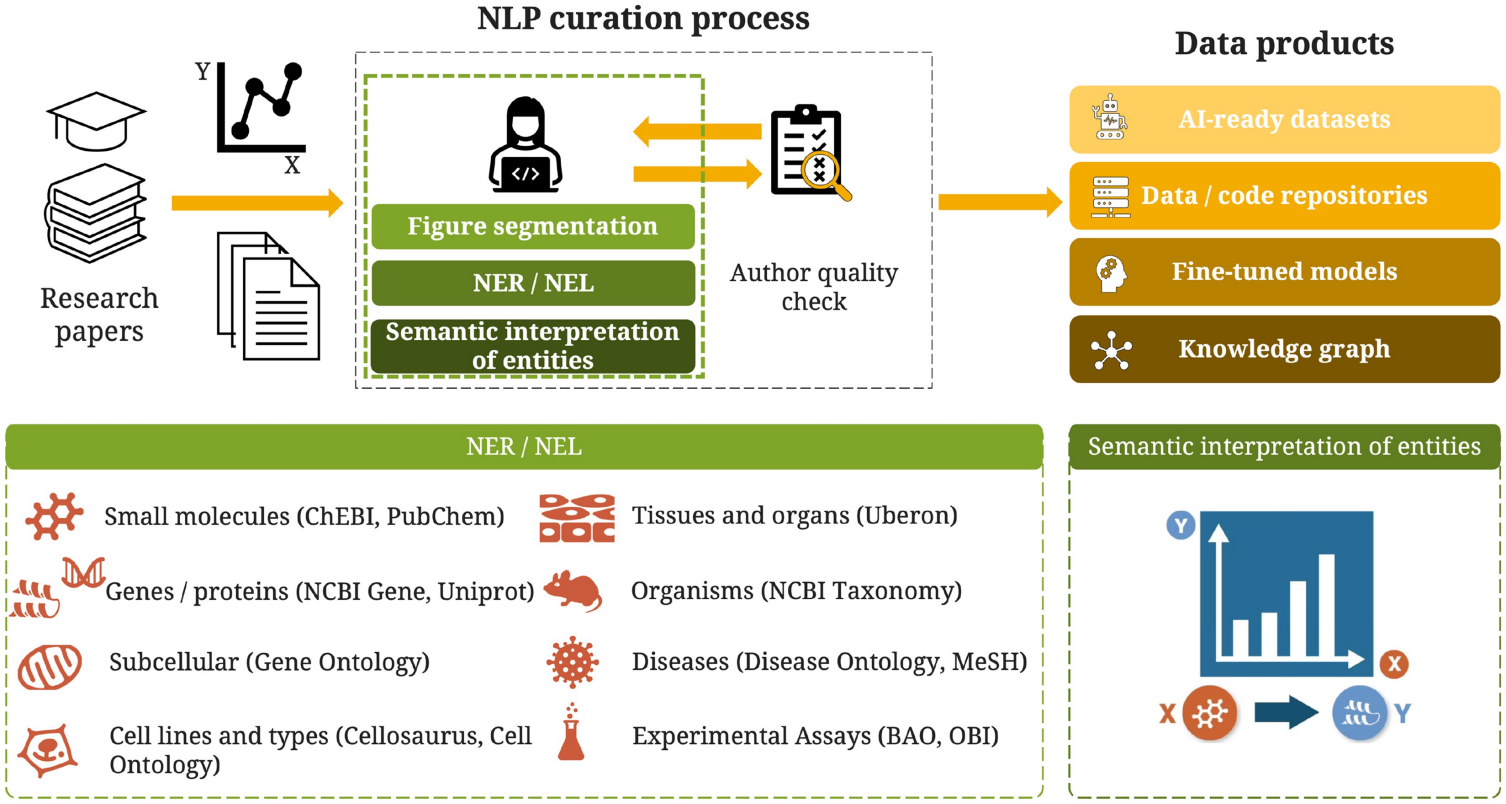
SourceData-NLP process for embedding article curation as part of the academic publishing process.

Since the SourceData annotation process is integrated into the publication workflow, it is strongly constrained by the nature of the editorial process in a scientific journal. Consequently, the annotation framework is designed to strike a balance between simplicity and expressiveness, ensuring feasibility while reflecting key elements of the experimental approach (Liechti et al. 2017). SourceData-NLP describes the experiments shown in scientific figures published in the field of cell and molecular biology. The annotations focus on biological entities relevant to the data’s scientific meaning and the underlying experimental design. More complex concepts, such as biological processes, pathways, or detailed entity attributes, such as post-translational modifications or other chemical conjugations, were excluded from the annotation schema to keep the process sufficiently simple. The annotation process is described in detail in Supplementary Appendix A, available as supplementary data at *Bioinformatics* online. It is performed by trained curators who have full access to the entire paper and are using a custom-made online curation tool while following the annotation guidelines provided in Appendix B, available as supplementary data at *Bioinformatics* online. The process includes the following steps (Fig. 1):

1. Splitting of composite figures into coherent panels (“figure segmentation”).
2. Tagging and linking of biological entities to external identifiers (related to the NE R and NEL tasks).
3. Categorization of entities into the role they play in the specific experimental design.

In brief, the annotation process starts with capturing all mentions of specific biomedical entities in the figure legend text. These bioentities represent biological “objects” that span organizational scales in living organisms, ranging from the molecular to the organismal level. The entities are accordingly classified into mutually exclusive “types”, including small molecules, gene products (genes and proteins), subcellular components, cell types, cell lines, tissues, organisms, and diseases (Fig. 1). Generic terms, such as “proteins”, “genes”, or “cells”, are excluded from the annotation process. Each entity is then linked to unique identifiers in external ontologies or knowledge bases to ensure unambiguous identification. For example, gene names are normalized to NCBI Gene identifiers, and proteins to UniProt identifiers. A complete list of the ontologies used can be found in Supplementary Table S1, available as supplementary data at *Bioinformatics* online.

While the primary focus of the SourceData curation process is on identifying discrete bioentities that represent biological “objects” (ranging from the molecular to the organismal scale), curators also annotate “conceptual” textual entities that provide important contextual information. These annotations include experimental assays, methodological approaches and disease classification, which add more details about the experimental setup and the medical significance of the reported scientific results.

The next step in the curation process is to determine the “role” of each bioentity within an experiment. In the following, for consistency with the guidelines provided to curators (Supplementary Appendix B, available as supplementary data at *Bioinformatics* online), we use “component” as a synonym for “bioentity” or “biological object”. The roles of biological components in an experiment include the following:

- **Measured variable**: A “component” (i.e. a bioentity) that is directly measured or observed.
- **Controlled variable**: Also referred to as perturbation, intervention, manipulation, alteration, or independent variable, this component is subjected to an experimental intervention. To be considered as a controlled variable, a bioentity must be altered in a targeted way, and the experiment must include a comparison across experimental groups designed to test its effect on the measured variable.
- **Experimental variable**: A component used to compare multiple experimental groups when no direct cause-and-effect relationship can be inferred between it and the measured variable.
- **Biological component**: A generic category for components relevant to the experiment but not fitting into the other roles. This often includes organisms, cells, or generic treatments present across all conditions.
- **Reporter component**: A proxy used to indirectly measure or observe a measured variable, typically part of a synthetic or engineered construct.
- **Normalizing component**: A component that is assayed to provide baseline measurements from each experimental group so that the data can be normalized across groups.

Annotating the roles of bioentities enables the representation of important information about experiments designed to test cause-and-effect relationships of the entities, while also categorizing other relevant entities appropriately. As such, the measured variable and the controlled variable are considered key components in describing an experiment. By definition, every experiment must have a measured variable, while a “controlled variable” can be absent. For example, purely observational experiments may only investigate the correlation between measured variables, without any controlled variables and thus without any causal interpretation of the results.

Determining the controlled variable involves identifying the entity that is subjected to an experimental perturbation and whose effect is compared to a control condition (see annotation guidelines for more details, Supplementary Appendix B, available as supplementary data at *Bioinformatics* online). For example:

- **Gene knockouts**: The genotype resulting from a targeted genetic disruption, or “gene knockout”, is compared to the wild-type genotype. Therefore, the targeted gene is the controlled variable.
- **Drug treatment experiments**: A drug-treated condition is compared to a mock treatment. Therefore, the drug is the controlled variable.

An “experimental variable” is a more generic role that applies to components that cannot be distinctly defined as either a measured or controlled variable. For example, if the expression of a gene product X is measured across several tissues, the measured variable is “gene product X”, whereas the tissues are annotated as “experimental variables”.

Given the molecular biology focus of the SourceData-NLP dataset, it was necessary to develop specific guidelines to address particular experimental types. For instance, agents used to induce conditional gene expression, such as doxycycline-mediated induction of engineered tetracycline-responsive transcription factor, are not annotated as controlled variables. Instead, the targeted gene is annotated as the controlled variable of interest.

### 3.2 Dataset validation and QC

**Figure 2.**
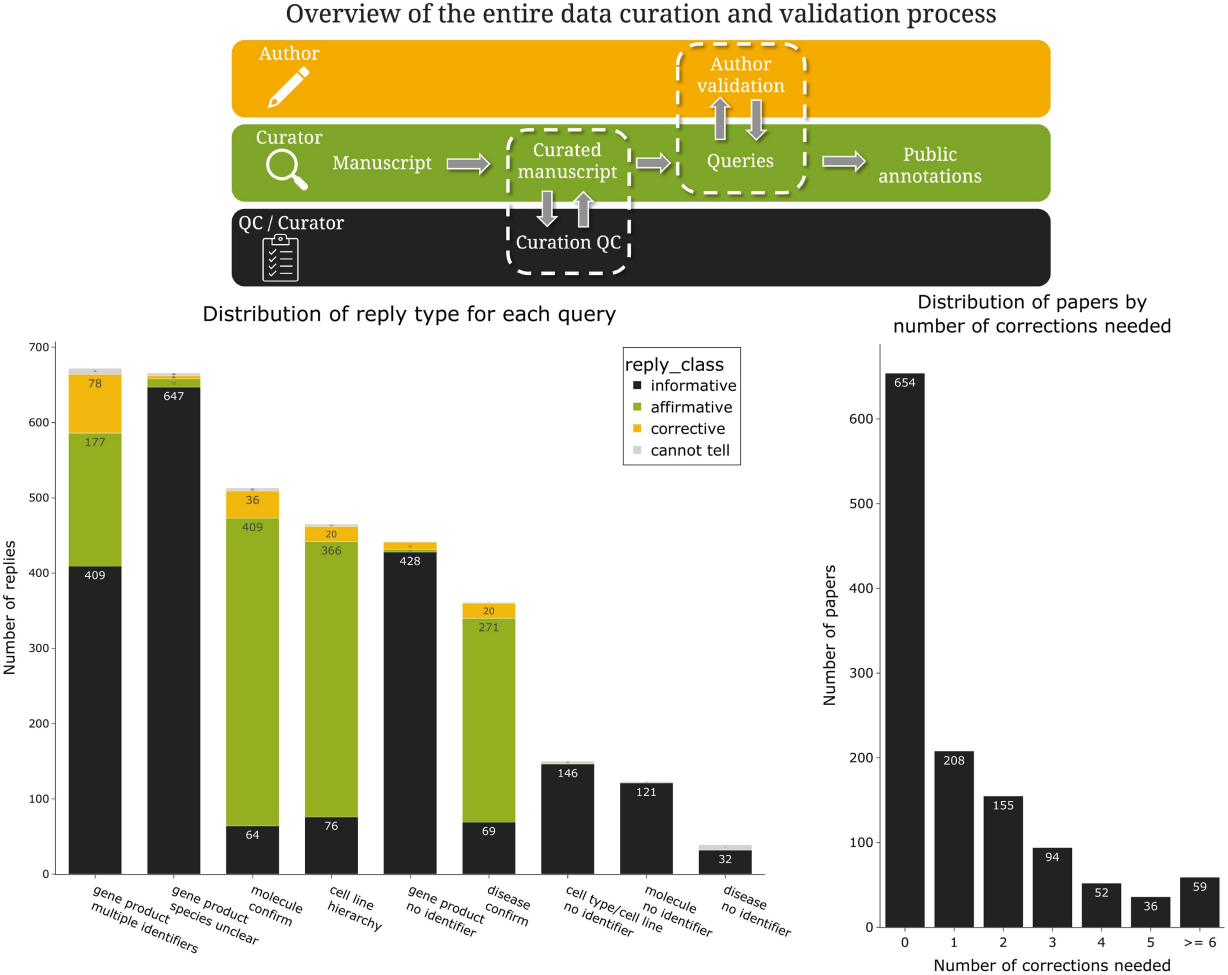
Overview of the data curation and validation feedback loops. *Top*: Schematic representation of the quality control (QC) workflow illustrating the bidirectional feedback mechanisms. The process involves three stakeholders: author (yellow), primary curator (green), and QC curator (black). Two distinct feedback loops are depicted: (i) the internal QC loop, wherein manuscripts failing validation are returned to the primary curator with specific correction requirements, and (ii) the author consultation (i.e. external) loop, wherein curators address standardized queries to authors to request clarification in case of uncertainties and unresolved ambiguities. *Bottom left*: Frequency distribution of manuscripts (*n* = 1258 for the period 2020–22) by required correction count following internal QC review. *Bottom right*: Distribution of query responses across nine primary query categories, stratified by response type: informative (black), affirmative (green), corrective (yellow), and indeterminate (gray). This quantitative analysis illustrates the functioning of the author feedback loop across different entity normalization challenges. The complete set of author queries is detailed in Supplementary Table S16, available as supplementary data at *Bioinformatics* online.

The curation workflow implements a two-step quality control (QC) process to improve annotation accuracy and reliability (Fig. 2). This process integrates both internal quality control procedures and external validation through author consultation.

#### 3.2.1 Internal quality control protocol

The first validation step consists of an internal QC performed by a secondary curator who examines the annotations made by the primary annotator (Fig. 2, top panel). This assessment follows evaluation guidelines (detailed in Supplementary Appendix B, available as supplementary data at *Bioinformatics* online). The QC protocol begins with a verification of fundamental annotation principles: each experimental panel must include at least one measured variable, and the experimental assay must be annotated to accurately reflect the corresponding figure content. The general evaluation guideline is to verify that “the core experimental hypothesis can be reasonably reconstructed using the captured tags, and whether the annotated entities effectively represent the performed experiment”. When discrepancies are identified, such as missing entities, superfluous annotations, or inconsistent relations, the QC curator conducts a more comprehensive assessment by examining the figure legend and, if necessary, the methods section to validate or reject the annotations. The manuscript is then returned to the original curator with specific recommendations for correction (Fig. 2, top panel). This internal feedback loop facilitates the identification and correction of systematic annotation errors, enhancing overall data quality.

Analysis of the correction patterns of 1258 manuscripts curated during the period from 1 January 2020 to 28 February 2022, revealed that most manuscripts (54.1%) required no corrections following the combined internal and external (explained below) validation processes, while 17.2% needed a single correction (Fig. 2, bottom left). Notably, 4.9% of manuscripts necessitated six or more corrections, indicating the presence of manuscripts with more substantial annotation challenges. This pattern suggests that while most annotations achieved high accuracy in their initial submission, a subset of manuscripts proved more challenging and required more extensive revision.

**Figure 3.**
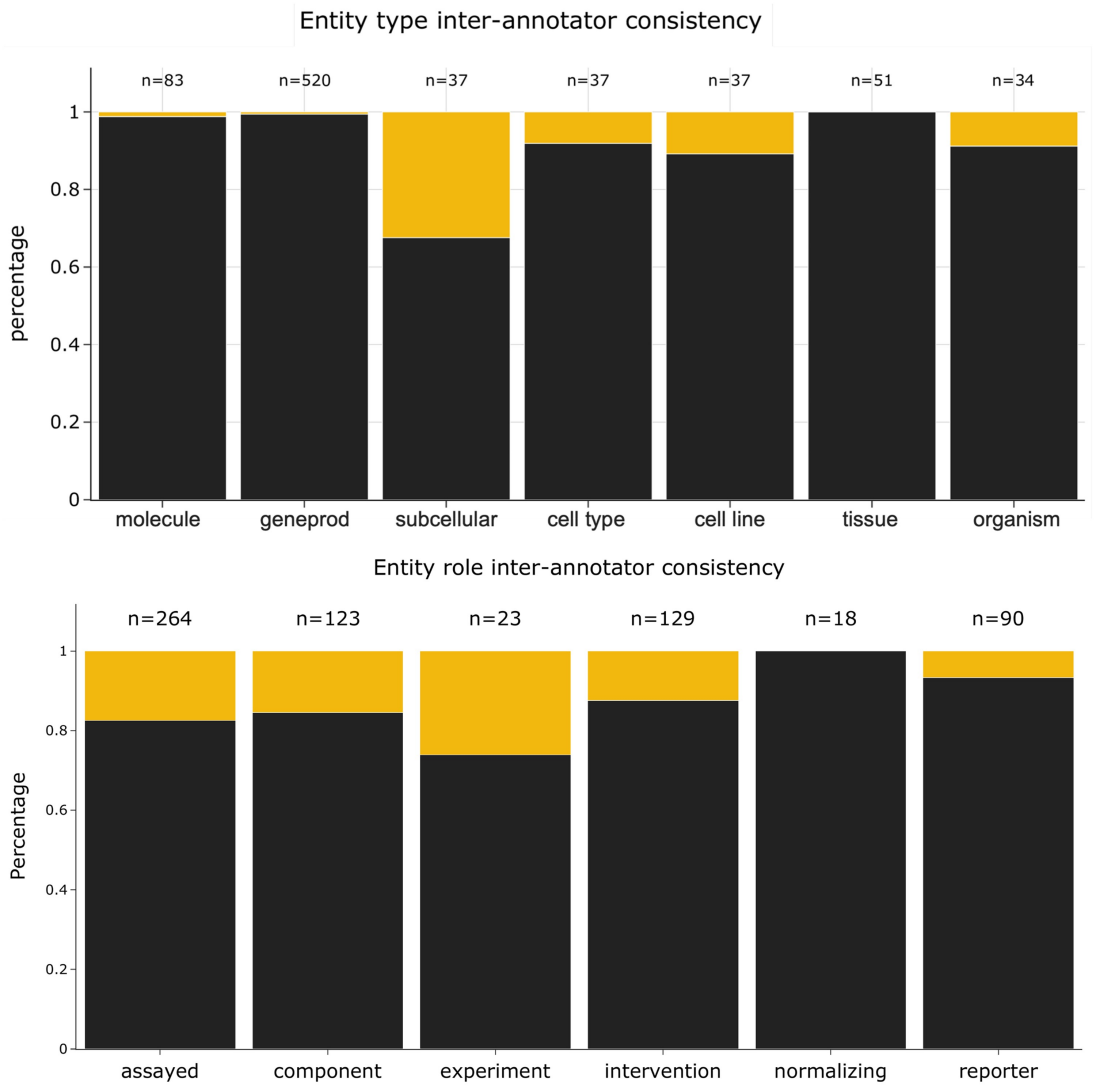
Inter-annotator agreement analysis. Top: Agreement percentages for entity category assignments across seven categories. Bottom: Agreement percentages for entity role assignments across six primary functional categories. For both panels, *n* values indicating the total number of entities in each bar.

Identifying bioentities and determining their role requires some understanding of the experimental design. To evaluate the difficulty of annotating bioentities types and their roles, a *post hoc* inter-annotator experiment was conducted on 100 panels from 10 randomly selected papers that were re-annotated by some of the authors (HS and TL, not previously involved in the primary annotation process). The resulting annotations, shown in Fig. 3 were then compared with those generated by the original curation team. Entity type assignment was reproducible with 99% agreement for “small molecules”, 99% for gene products, 92% for cell types, 89% for cell lines, 100% for tissues and organs and 91% for species and organisms. Entity role assignments demonstrated high consistency for the roles “controlled variable” (86.0%), “measured variable” (83.3%), and “generic component” (84.6%), “normalizing component” (100%) and “reporter” (91.1% agreement). Assignment of the role “Experimental variable” was the most variable (72.7%). This role is assigned when it is not possible to infer a cause-and-effect relationship between the assigned component and the measured variables of the experiment, as described in the curation guidelines where examples are provided. This role therefor covers cases where the role of an entity can be challenging to interpret and therefore create more difficulties for curators.

#### 3.2.2 Author validation process

The second validation step is an external validation phase, wherein annotations are shared with the authors through a dedicated validation interface. This platform enables authors to:

1. Respond to specific queries from the curators in cases of annotation uncertainty
2. Provide free-text feedback on any annotations

The curator-author interaction is helpful to resolve normalization uncertainties. For example, a frequently employed query solicits clarification regarding the species origin of experimental proteins: “We were uncertain about the concrete species of origin of the protein and therefore could not assign a UniProt ID. Kindly help us find the most accurate identifier for this entity. A species of origin and/or reference number from a vendor if appropriate would also be helpful.” The complete set of templated queries used in the author-feedback process is detailed in Supplementary Table S16, available as supplementary data at *Bioinformatics* online.

Authors were allotted a two-week period to respond to queries, with their feedback subsequently incorporated by one of the original curators. To provide an overview of this process, we retrospectively analyzed manuscripts curated during the period 2020 to 2022 and classified response types into four categories: informative (providing specific identification details), affirmative (confirming curator suggestions), corrective (modifying curator annotations), and indeterminate (insufficient information for resolution). This classification system enables systematic analysis of the author feedback mechanisms and their contribution to annotation quality (Fig. 2, bottom right). The distribution of query responses across eight primary query categories reveals varying patterns of author engagement, with gene product queries involving multiple identifiers and those with unclear specifications being the most frequent categories that require consultation.

### 3.3 Dataset summary

The SourceData-NLP dataset comprises 18 689 figures, segmented into 62 543 annotated panels derived from 3223 articles published across 25 journals. Given the integration of the curation process into EMBO Press’ publishing workflow, the dataset predominantly features papers from EMBO Journal, EMBO Reports, Molecular Systems Biology, and EMBO Molecular Medicine (89.7% of the corpus, *n* = 2891 papers). The dataset encompasses 801 818 entity annotations, of which 686 846 (85.7%) are linked to standardized identifiers in external reference databases. Table 1 presents the comprehensive distribution of these entities across annotation classes.

**Table 1.** Comprehensive overview of annotated bioentities in the SourceData-NLP dataset.^a^

		NER			Roles			NEL	
				Controlled	Measured				
	Total	Unique	%	Intervention	Assayed	Other	Total	Unique	%
Small mol.	93 582	9089	9.71	45 517	15 350	32 715	87 512	4776	5.46
Gene products	355 433	29 001	8.16	108 577	158 048	88 808	286 561	29 244	10.2
Subcellular	44 231	3932	8.89	1243	29 218	13 770	40 109	911	2.27
Cell type	31 336	2597	8.28	754	13 201	17 379	31 336	558	1.78
Cell line	30 457	1724	5.66	266	6647	23 544	30 441	1064	3.50
Tissue	40 677	4356	10.71	578	11 959	28 140	37 381	1437	3.84
Organism	48 589	2042	4.20	3278	9082	36 229	48 050	700	1.46
Disease	8045	1280	15.9				7430	567	7.63
Exp. Assay	149 468	13 056	8.73				118 026	740	0.63
Total	801 818	67 077	8.37	160 213	243 505	240 585	686 846	39 997	5.82

a This table presents a detailed breakdown of annotations, categorizing them by entity type for NER and the roles assigned within experimental contexts. Additionally, it provides statistics for NEL, including unique mentions tied to external identifiers. Percentages indicate the uniqueness of entity mentions within each category, reflecting the diversity of the dataset’s contents.

**Figure 4.**
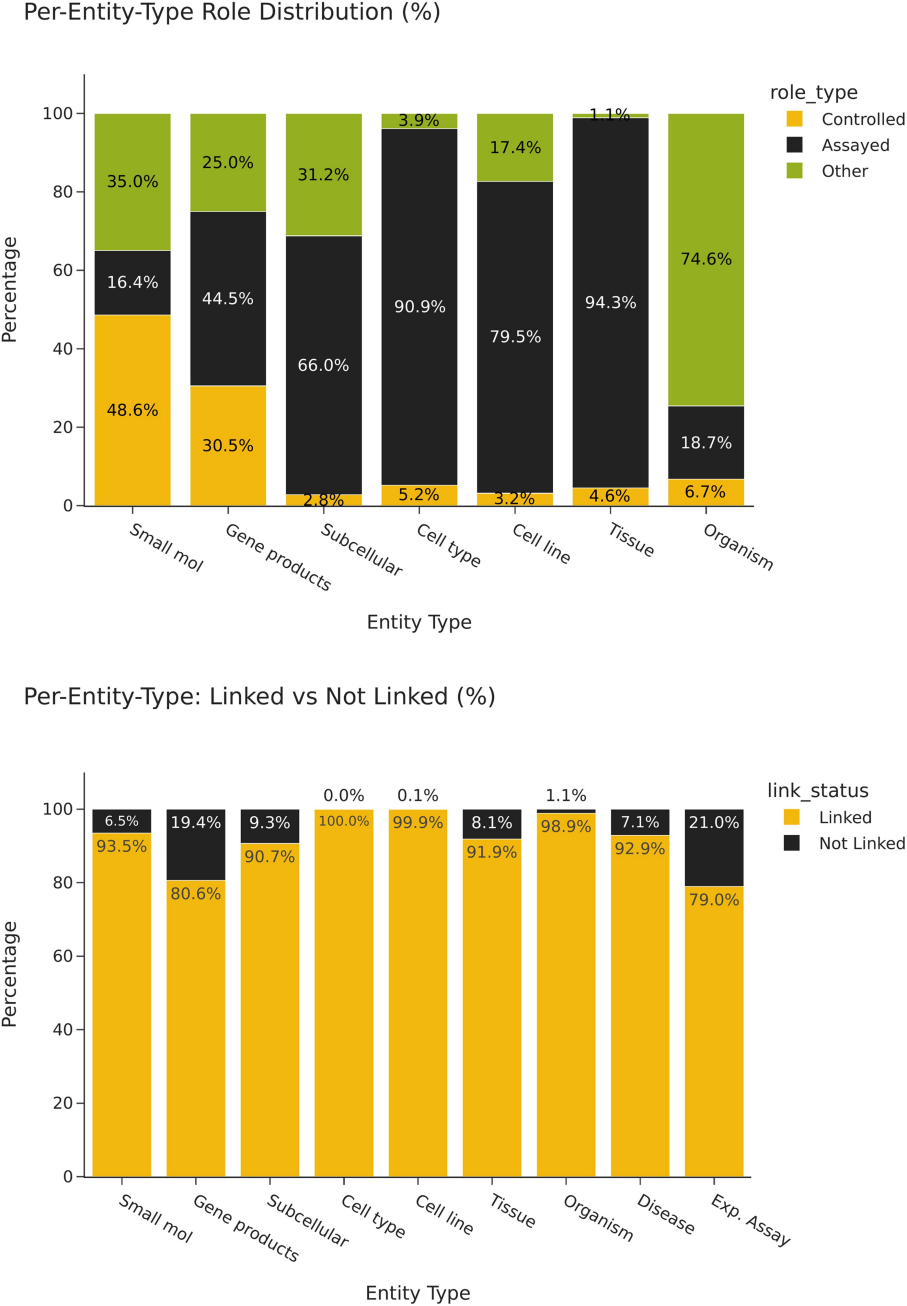
Distribution of linked entities and experimental roles. Top: A stacked bar chart showing the distribution of roles (Controlled, Assayed, Other) per entity type. Each bar is subdivided by the proportion of entities that fall under each role category, expressed as a percentage. Bottom: A stacked bar chart illustrating the proportion of linked versus not-linked entities for each entity type, again expressed as a percentage.

Gene products constitute the predominant entity class, comprising 355 433 annotations (44.3% of all entities) corresponding to 29 001 unique entities. As illustrated in Fig. 4 (top panel), the distribution of experimental roles varies substantially across entity types, reflecting their distinct functional roles in experimental designs. Gene products demonstrate a relatively balanced distribution across roles, with 30.5% functioning as controlled variables, 44.5% as measured variables, and 25.0% in other capacities. This balanced representation makes gene products particularly suitable for role classification tasks, as demonstrated in Section 2.4.2.

Small molecules exhibit a markedly different distribution pattern, with 48.6% serving as controlled variables (frequently corresponding to drug treatments or chemical perturbations) and only 16.4% as measured variables. In contrast, cell types, cell lines, and tissues predominantly appear in supporting roles rather than as experimental variables, with only 5.2%, 3.2%, and 4.6%, respectively functioning as controlled variables. This predominance of supporting roles aligns with their typical function in experimental designs, where they often constitute the biological context rather than the primary variables under investigation.

The difference between NER and NEL entity counts (114 972 entities without external identifiers) merits explanation. As depicted in Fig. 4 (bottom panel), the proportion of entities successfully linked to external identifiers varies substantially across entity types. While certain classes demonstrate high normalization rates (e.g. cell types at 100%, organisms at 98.9%), others exhibit lower rates of successful linking (e.g. gene products at 80.6%, experimental assays at 79.0%). These disparities stem from multiple factors: (i) prioritization of entity linking for measured and controlled variables over supporting entity roles, (ii) inherent ambiguity in certain entity mentions that preclude definitive normalization, and (iii) the absence of suitable reference identifiers in the selected ontologies and knowledge bases for some specialized entities.

Disease entities represent a relatively small proportion of the dataset (8045 annotations, 1.0% of total), reflecting their later inclusion in the annotation schema during the project’s evolution. Initially, the project scope focused primarily on molecular and cellular components, with disease annotations incorporated subsequently to enhance the dataset’s clinical relevance.

To our knowledge, SourceData-NLP represents one of the most comprehensive biomedical annotated datasets currently available for NER and NEL tasks in the molecular biology domain. To facilitate reproducible research, the dataset has been segmented into training, validation, and test partitions following an 80–10-10 ratio stratified by source publication to prevent information leakage between partitions.

### 3.4 Finetuning language models for downstream NLP applications

#### 3.4.1 Named-entity recognition

To illustrate the utility of the SourceData-NLP dataset, we evaluated the performance of two prominent biomedical pre-trained language models, PubMedBERT and BioLinkBERT, in the context of the NER task. We compared the base (110 M parameters) and large (330 M parameters) versions of these models. The models were fine-tuned on the SourceData-NLP dataset using a multi-class training approach, where a single model assigns mutually exclusive labels across all entity categories. Details on the fine-tuning procedure can be found in the Supplementary Material C.1, available as supplementary data at *Bioinformatics* online.

The results of this experiment, summarized in Table 2, demonstrate regular incremental improvements with the larger versions of the models, while also revealing each model’s intrinsic variability via their standard deviations. Among the tested models, BioLinkBERT yields the highest overall F1 performance, but large PubMedBERT exhibits the lowest standard deviation across most entity classes, indicating that it is more stable in its predictions. For instance, the large versions typically, but not always, outperform the base models across most entity categories (e.g. BioLinkBERT shows a notable 4.89% improvement in the Disease category). In some categories, the large models exhibit wider fluctuations (e.g. the standard deviation for “Experimental Assay” in large BioLinkBERT is ±6.47). This suggests that while more powerful architectures usually boost performance, they can also yield higher variability in certain entity classes—particularly those with limited training examples or more complex nomenclature.

**Table 2.** Performance comparison of base and large model variants on the NER task.^a^

**Model**		PubMedBERT			BioLinkBERT		Support
Model size	base	large	Δ(%)	base	large	Δ(%)	
Gene product	91.9 ± 3.35	92.3 ± 0.10	0.43	89.2 ± 1.01	**92.7 ± 2.65**	3.78	26 321
Cell line	89.8 ± 0.92	90.4 ± 0.98	0.66	90.7 ± 0.55	**90.8 ± 0.76**	0.11	2367
Organism	86.7 ± 0.96	**87.8 ± 0.45**	1.25	87.7 ± 1.05	87.5 ± 0.22	−0.23	4222
Small molecule	84.8 ± 3.08	85.0 ± 0.31	0.24	82.9 ± 0.63	**86.2 ± 3.34**	3.83	6932
Tissue	82.6 ± 2.19	**83.5 ± 0.89**	1.08	82.9 ± 1.36	83.4 ± 0.92	0.60	3851
Subcellular	78.8 ± 3.18	79.4 ± 0.21	0.76	**79.6 ± 0.85**	79.4 ± 2.41	−0.25	4671
Cell type	72.1 ± 2.11	**72.6 ± 0.39**	0.69	71.4 ± 0.58	72.2 ± 0.54	1.11	2872
Disease	66.6 ± 5.36	68.6 ± 0.72	2.92	66.1 ± 1.58	**69.5 ± 0.64**	4.89	602
Exp. Assay	67.3 ± 3.77	67.8 ± 3.61	0.74	67.1 ± 2.17	**68.7 ± 6.47**	2.33	11196
**Micro avg.**	83.7 ± 2.87	84.2 ± 0.66	0.59	82.5 ± 0.60	**84.7 ± 2.48**	2.60	**63** **034**
**Macro avg.**	80.1 ± 2.72	80.8 ± 0.38	0.87	79.7 ± 0.50	**81.2 ± 1.41**	1.85	**63** **034**
**Weighted avg.**	83.6 ± 3.00	84.2 ± 0.57	0.71	82.4 ± 0.61	**84.6 ± 2.18**	2.60	**63** **034**

a The results show the average F1 scores, and their standard deviations, obtained through five inference rounds. The column quantifies the performance gain of the large models over their base counterparts. The support column indicates the number of mentions of the given entity class in the test dataset. For each entity, the best performance is highlighted in bold.

Overall, comparisons across entity classes reveal that performance varies significantly: gene products, cell lines, organisms, and small molecules achieve higher F1 scores, whereas subcellular components, cell types, diseases, and experimental assays perform less consistently. Notably, these latter categories tend to have more complex or varied terminologies (e.g. “endoplasmic reticulum” or “hippocampal CA1 pyramidal neuron”), which likely contribute to both lower average F1 scores and higher standard deviations. Conversely, entities such as gene products and cell lines (e.g. “Creb1” or “HeLa”), which have simpler names and appear more frequently, exhibit more stable performance with smaller standard deviations. Taken together, these findings underscore the value of larger models in capturing richer patterns across diverse biomedical entities, while also highlighting that certain categories remain challenging and prone to higher variability in model predictions.

The relative contribution of memorization versus generalization in NER transformer models is not fully understood. Previous work by Kim and Kang (2022) shows that models may not generalize well because they tend to exploit dataset biases or substandard naming conventions. In contrast, Tänzer *et al.* (2021) suggests that models can be robust to noisy data, assuming the patterns they learn are sufficiently frequent. To investigate these two aspects of the learning process, we evaluate the memorization and generalization capabilities of PubMedBERT and BioLinkBERT, both of which are fine-tuned on the SourceData-NLP dataset. Following Kim and Kang (2022) we divided test set entities into “memorization” and “generalization” subsets. Entities were considered memorized if they were encountered during fine-tuning training or if they were present in the validation set. In contrast, the generalization subset is exclusively composed of entities that the model has never seen in either the training or the validation set.

**Figure 5.**
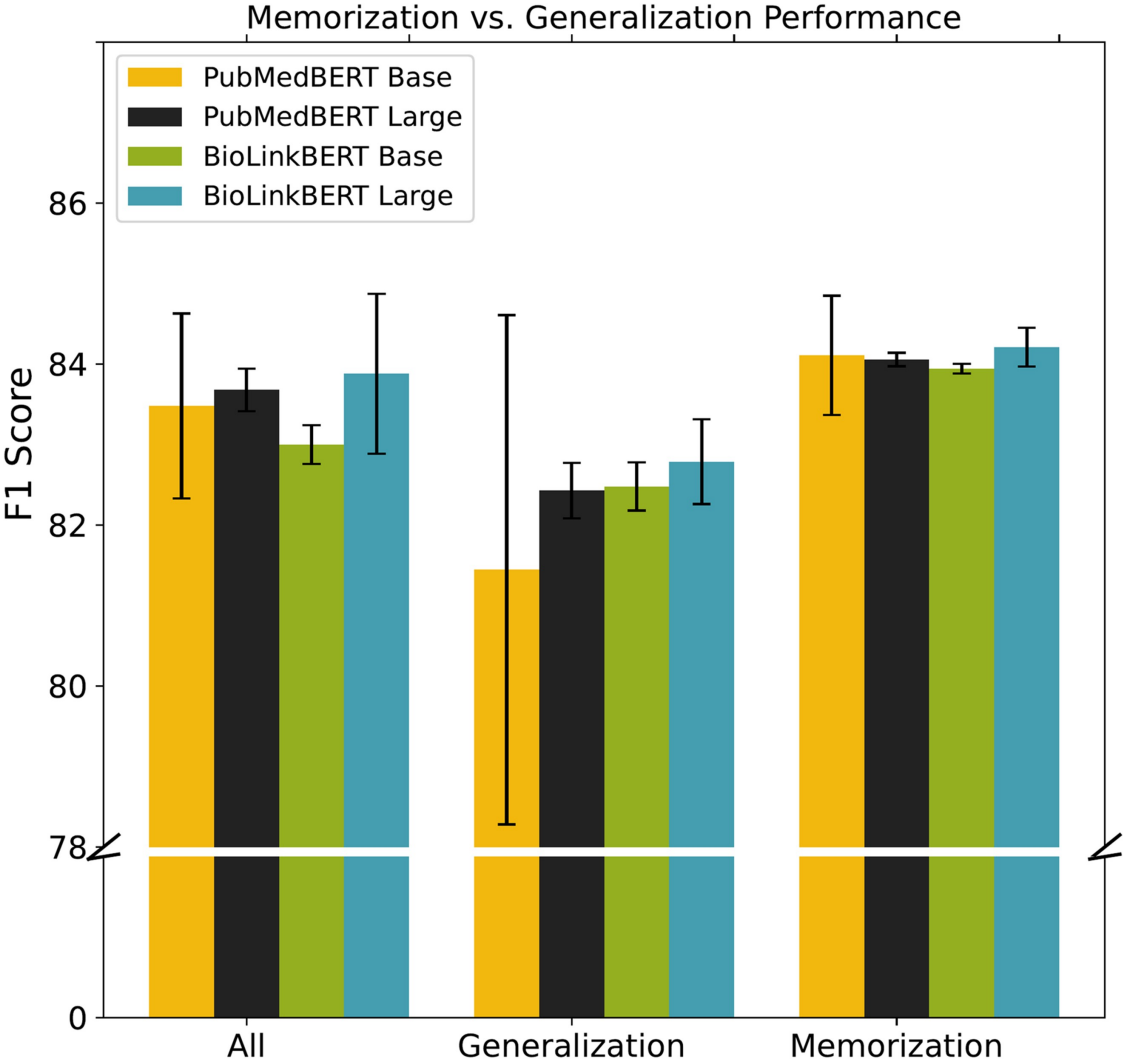
Memorization versus generalization performance of PubMedBERT and BioLinkBERT models. The bar chart compares the F1 scores, distinguishing between overall performance, and specific performance in memorization and generalization tasks. The error bars show the standard deviation of the F1 scores over five inference runs. The colors indicate PubMedBERT Base (yellow), PubMedBERT Large (black), BioLinkBERT Base (green), and BioLinkBERT Large (blue).

The results are shown in Fig. 5. The findings reveal that PubMedBERT and BioLinkBERT achieve higher F1 scores on entities from the memorization subset than those from the generalization subset. Intriguingly, the base version of PubMedBERT shows slightly better memorization than its larger counterpart, which may indicate some overfitting in the larger model. BioLinkBERT exhibits superior generalization with novel entities relative to PubMedBERT, indicating that its initial training regimen may have enhanced its ability to handle unseen data effectively.

To improve the models’ generalization capabilities, particularly for rare entity types like subcellular components and diseases, where performance is currently lower, several approaches could be explored. We initially conducted a series of experiments employing stochastic entity masking during the fine-tuning phase, wherein biomedical named entities were systematically replaced with [MASK] tokens at probabilistic rates ranging from *P* = .02 to *P* = .5 across multiple experimental trials. This methodological intervention was based on the hypothesis that forced contextual inference would enhance generalization capabilities by reducing reliance on entity-specific lexical memorization. Subsequent quantitative analysis, however, revealed a trend of lower F1 scores (see Supplementary Fig. S16, available as supplementary data at *Bioinformatics* online) for previously unseen entities across all experimental conditions, suggesting that alternative approaches to generalization enhancement may be required for rare biomedical entity recognition tasks.

#### 3.4.2 Semantic interpretation of experimental roles

Within the context of the SourceData-NLP project, we introduced a novel NLP task aimed at determining the experimental roles of entities in biomedical experiments. This token classification task assigns the “measured variable” or “controlled variable” roles to entities based on context. In this task, entities without one of these two roles are assigned the “none” category. This task is challenging, even for curators, as it requires interpreting the experimental design and is highly dependent on contextual information. We were therefore interested in investigating to what extent this task can be learned from the SourceData-NLP dataset.

Our experiments utilized both base and large versions of PubMedBERT. We primarily focused on gene products due to their balanced representation in the dataset (42% as the “controlled variable” and 58% as the “measured variable”).

We explored three distinct approaches for training:

1. **Context-only approach**: all gene products tagged in the SourceData-NLP dataset were first masked. The pre-trained models were then fine-tuned to classify these masked entities into “measured variable”, “controlled variable” or “none”. This approach ensures that the models learn the classification task solely based on context, without being influenced by the identity of the specific entities.
2. **Marked-entity approach**: gene products were first flanked by a special token to mark their position in the text explicitly. The pre-trained models were then fine-tuned without any entity masking. This allows the models to learn from both the context and the identity of the entities.
3. **Single-step approach**: in contrast to the previous approaches, here entities were not masked, and their position was not marked. The models were therefore trained to both recognize the entity and classify its role in a single step, thus combining NER with semantic role classification in a joint task.

In the inference stage, a prior NER step is required to identify entity mentions in the text for both context-only and marked-entity tasks. However, the single-step approach handles both tasks simultaneously, eliminating the need for a multi-stage pipeline workflow.

The results of these experiments, detailed in Table 3, validated the efficacy of our novel approaches. Particularly, the models employing the marked-entity and context-only approaches demonstrated robust capabilities in accurately identifying the nuanced roles of bioentities within complex experimental setups. Since these first two approaches require a prior NER step during inference, we also report a combined score for the pipeline by multiplying the F1 scores of the role classification step by the respective NER scores, as detailed in Table 2. The corrected F1 scores across different approaches ranged from 81.4% to 84.2%, indicating the potential for high accuracy in the contextual interpretation of biomedical entities. This highlights the unique capability of SourceData-NLP for accurately representing causal hypothesis testing in literature.

**Table 3.** Micro averaged F1 scores of the experimental role of entities task for gene products and small molecules, using the PubMedBERT (PMB) and BioLinkBERT (BLB) models.^a^

Entity class	Model	Context only	Corrected	Marked-entity	Corrected	Single-step
GENEPROD	PMB-base	91.2	83.8	91.6	84.2	81.4
	PMB-large	91.0	84.0	92.3	85.2	82.5
	BLB-base	92.1	82.2	91.7	81.8	82.4
	BLB-large	77.9	72.2	**92.4**	**85.7**	80.2
SMALL MOL	PMB-base	54.6	46.3	96.1	81.5	76.9
	PMB-large	61.9	52.6	95.7	81.3	79.6
	BLB-base	94.9	78.7	96.1	79.7	76.6
	BLB-large	53.8	46.4	**96.7**	**83.4**	79.6

a The table shows scores for three approaches: context-only, marked-entity, and single-step. For context-only and marked-entity approaches, both uncorrected scores (role classification only) and corrected scores (combined with NER performance) are shown. Corrected scores ('corrected' columns) are calculated by multiplying the role classification F1 scores by their respective NER F1 scores from Table 2, representing the performance of the complete pipeline. The single-step approach inherently combines NER and role classification, thereby eliminating the need for correction. The best performance obtained with gene products and small molecules, respectively, are indicated in bold.

Additionally, we extended these experiments to small molecules (shown in the lower block of Table 3), confirming that the pipeline’s performance remains strong across different entity classes. In both gene products and small molecules, the “marked-entity” approach consistently yields the best results, with BioLinkBERT-large achieving the highest corrected F1 scores of 85.7% and 83.4%, respectively. Interestingly, we observed that while gene products demonstrate relatively stable performance across different approaches, small molecules show more pronounced variability—particularly in the context-only approach, where performance fluctuates significantly between model variants (53.8%–94.9%). This suggests that entity identity may play a more crucial role in role classification for small molecules than for gene products.

We did not extend this experiment to other entity types due to their substantially lower representation and extreme role imbalance in the dataset (see Table 3 and Fig. 4). For instance, subcellular components appear as controlled variables in only 2.8% of cases, while cell types, cell lines, and tissues exhibit even greater imbalance (5.2%, 3.2%, and 4.6% as controlled variables, respectively). Such distributional asymmetry would introduce significant statistical bias in model training and evaluation. Future work could address this limitation through targeted data collection or specialized data augmentation strategies for underrepresented entity-role combinations.

#### 3.4.3 Multimodal segmentation of compound figures

We used the SourceData-NLP dataset to generate a dataset for assembling a multimodal pipeline that separates compound figures into their constituent panels and matches them to the corresponding panel captions. We followed a two-step procedure to achieve this goal. First, we used object detection algorithms to separate the figure into its panels. Second, we employed a multimodal LLM to extract the corresponding panel description from the figure caption, ensuring that the panel caption is understandable on its own without requiring the context of the full figure caption.

To evaluate our panel segmentation approach, we created a dataset of 13 039 figures with annotated panel boundaries, split into training (11 735), validation (651), and test (653) sets. We fine-tuned a pre-trained YOLOv10 model (Wang *et al.* 2024) using a distributed multi-GPU setup with automatic batch size optimization and standardized 720-pixel image dimensions (see Supplementary Appendix C.2 for training details, available as supplementary data at *Bioinformatics* online). Model performance was assessed using two metrics: mAP_50_ (mean average precision with 50% intersection-over-union threshold) and mAP_50-95_ (average precision across IoU thresholds from 50% to 95% in 5% increments). The fine-tuned model achieved mAP_50_ = 98.2% and mAP_50-95_ = 87.0% on the test set, which contained 3067 panels, with 52 false negative errors.

We conducted a detailed error analysis of the 52 panels that were not correctly identified by the object detection model (see Supplementary Appendix C.2 for representative examples, available as supplementary data at *Bioinformatics* online). Overall, plots and charts showed higher detection accuracy compared to image-based panels, which may be attributed to sampling bias in our training data. The most frequent errors occurred with composite panels containing multiple subpanels (14 cases), where the model generated excessive detection reports. Other error categories included partially overlapping panels (9 cases), undetected Western blot images (6 cases), panels with non-rectangular boundaries (2 cases), and panels with interconnecting schematics (2 cases). The remaining cases showed no clearly identifiable pattern; however, we observed two possible trends: panels positioned at the bottom of figures had a higher likelihood of misidentification, and detection accuracy decreased with less conventional panel arrangements that deviated from standard, grid-like layouts.

To map each segmented panel to its corresponding part of the caption, we utilized API calls to the OpenAI GPT-4o multimodal model. Each API call contained the text of a full figure caption and the image of one of the panels of the corresponding figure extracted with our image segmentation model described above. The results returned by the model are the extracted panel description, minimally edited to be fully understood without the need for context from the full figure caption. The system prompt used for the LLM is shown in Supplementary Appendix C.2, available as supplementary data at *Bioinformatics* online. The accuracy of this second step for caption-panel matching is 97.4%. Here, we consider a correct result every panel assigned the correct label, while every panel with a false label assignment is considered a miss. The accuracy is measured only on those panels correctly identified by the object detection algorithm.

To contextualize our results with related work in scientific figure analysis, we examined performance reported on the ImageCLEF2016 dataset. Prior work on this dataset achieved accuracies of up to mAP_50_ = 90% and mAP_50-95_ = 72% (Jiang *et al.* 2021) by finetuning YOLOv3 (Adarsh *et al.* 2020). However, direct comparison with these works is not possible because they were based on the medical task of ImageCLEF2016 (de Herrera *et al.* 2016), which focuses on extracting images from scientific figures, whereas we extract the entire panel, including the image, labels, and analytical plots.

## 4 Discussion

The SourceData-NLP shows the benefits of integrating curation within the publishing process. Through our figure-centric annotation approach, we have produced one of the most extensive NER and NEL datasets currently available in the field of molecular biology and life sciences. The scale and utility of SourceData-NLP for training large language models underscore the potential of this approach to enhance the accessibility and reusability of scientific data, while also accelerating the development of AI tools for scientific discovery.

By focusing the annotations on figures, rather than processing papers in their entirety, the curation effort was manageable and could be sustained over an extended period. Moreover, by concentrating on figures, we created a unique multimodal dataset that links the images of individual experimental results shown in figure panels to the corresponding segments of their captions. This structure enables the exploration of novel AI tasks, such as figure-caption matching, image-based information retrieval, and multimodal vector representations. We recognize, however, that tables and supplementary materials often contain substantial additional evidence. Future expansions of our approach could incorporate these supplementary elements to provide a more comprehensive coverage of experimental evidence.

The SourceData-NLP contains more than 600 000 disambiguated entities present in 62 543 annotated figure panels from 3223 papers, making it a useful resource for training NLP models. Its diverse representation of biomedical entities ensures that resultant models are adept at addressing challenges inherent to the field. To facilitate access for researchers to SourceData-NLP, we provide scripts to obtain and process the raw data from the SourceData API into both XML and graph database (neo4j) formats, and from these into machine-learning-ready formats.

Additionally, we have delineated a novel NLP task centered on interpreting the role of entities within a given experimental design. We show that this task, which depends heavily on contextual information, can be efficiently learned to determine whether a gene product is measured (a “measured variable”) or whether it is the target of an experimental perturbation (a “controlled variable”). The discrimination of controlled and experimental variables is a unique property of SourceDataNLP.

In biomedical research, testing causal hypotheses is a key experimental approach to investigating the molecular mechanisms underlying biological processes and human diseases (for example: “does the perturbation of gene product X influence the measurement of gene product Y?”). The models trained on SourceData-NLP should therefore assist in the large-scale extraction of causal hypotheses from the literature and the traceability of their links to published scientific results. While our approach does not address comprehensive relationship extraction (e.g. protein–protein interactions or regulatory pathways), it does capture the fundamental experimental roles of these entities within the scope of an experiment. This functional characterization underpins numerous downstream applications such as knowledge graph construction, hypothesis generation, and literature-based discovery (Wei *et al.* 2019).

Over the years, significant efforts have been made to create annotated datasets for training and benchmarking algorithms on NER and NEL tasks (see bigBio Fries *et al.* 2022 for a detailed summary of these efforts). The recent BioRED dataset (Luo *et al.* 2022) has a similar scope to SourceData-NLP. It annotates genes, diseases, chemicals, genetic variants, species, and cell lines. However, BioRED is focused on the relation detection (RD) task, which reveals interactions between entities. BioRED has a set of more than 20 000 entities tagged with links to databases and includes a novelty classification label intended to highlight novel discoveries. Another recently published dataset is the Europe PMC Annotated Full-text Corpus (Yang *et al.* 2023). They report a total of over 72 000 annotated entities of the classes gene/protein, disease, and organism. Their data sample covers isolated sentences from the 300 selected full-text articles. The annotations were done using the triple-anonymous annotation procedure, with no involvement of the authors of the manuscripts. With these properties, the scope of the Europe PMC Annotated Full-text Corpus differs from that of the SourceData-NLP dataset.

Previous studies typically focus on accurately extracting entities (NER/NEL) and their relationships by annotating sentences, abstracts, or full texts. The unique advantage of SourceData-NLP lies in its innovative approach to annotating figure captions that describe scientific results and in defining the roles that biomedical entities play within specific experimental designs. Moreover, SourceData-NLP encompasses a wider set of entity classes, offering more comprehensive coverage that enhances the dataset’s utility across various biomedical research domains. Finally, the SourceData-NLP dataset is inherently multimodal, as the annotated text is paired with its corresponding scientific figures and panels.

To the best of our knowledge, previous work has not extensively explored such detailed annotation of experimental roles of bioentities in scientific literature, along with the expansive array of tagged entity classes and a multimodal format. This novel aspect of SourceData-NLP facilitates the creation of data models that capture critical features of scientific experiments in biomedical research, thereby providing a deeper and more functionally relevant layer of data that enhances the potential for breakthrough discoveries in the field.

### 4.1 Limitations

One limitation of the SourceData-NLP dataset is the inherent noise of human-based curation. The constraints of the publishing process did not allow curation by multiple annotators. Even though an independent validation step by authors and quality control by the editorial office were in place, all discrepancies could not be eliminated. While the SourceData-NLP is already a robust resource, we anticipate that the workflow will benefit from enhanced automated consistency checks.

The distinction between “measured variables” and “controlled variables” represents a first step in capturing the causal hypotheses that underlie an experiment. In our experience, within the scope of a single experiment, as shown in an individual panel, the co-occurrence of “controlled variable” and “measured variable” entities is often a reasonable approximation for extracting the list of hypotheses tested in the experiment. It remains, however, an approximation since our annotations do not capture the full structure of the experimental groups. In addition, the actual hypotheses as formulated by the authors often include expectations about the direction (inhibition or stimulation) of the hypothesized effect. Methods dedicated to relationship extraction and deep understanding would be required to extract full descriptions of causal hypotheses from manuscripts.

The scope of the journals from which the dataset was generated is focused on cell and molecular biology. While it provides a rich resource for entities such as small molecules, genes, proteins, subcellular components, and cell lines, its coverage of other biomedical entities, including cell types, tissues, organs, and diseases, is sparser. Our approach is therefore mostly relevant for molecular and cell biology. It remains open to see how applicable and useful our approach would be in fields such as ecology, evolution, and neurosciences. For medically and clinically oriented fields, phenotypes, diseases, biological and physiological processes and states might be more appropriate than the simple bioentity-oriented approach chosen here.

Our dataset is clearly not balanced in terms of the frequency of the different entity types and their roles, as shown in Table 1 and Fig. 4. As such, the dataset will reflect the biases of papers accepted by the journals used to build the dataset. This is inherently linked to the workflow used in this study, which is to annotate accepted papers during the editorial process, without the possibility of balancing the data.

### 4.2 Future work

Including medical and clinical papers or collaborating with journals outside of molecular biology would be a possible approach to mitigate the current bias toward molecular and cell biology. An additional promising approach to overcoming some of the limitations above is to utilize data augmentation strategies during model training. One possibility is to use generative models, which produce synthetic examples from an exhaustive entity dictionary. These synthetic examples could considerably expand the dataset to improve the coverage, mitigate biases and robustness of fine-tuned models (e.g. Guo *et al.* 2023, Whitehouse *et al.* 2023, Yuan *et al.* 2023). An alternative strategy is to use existing examples as templates in which a fraction of the annotated entities could be replaced by sampling terms of the appropriate type from dictionaries or controlled vocabularies in a process akin to ontology-guide data augmentation (Abdollahi *et al.* 2020). This approach would provide a richer and more varied set of examples, potentially enhancing the dataset’s depth. A third approach to this problem could be to merge SourceData-NLP with other NER datasets dedicated to the different classes annotated in SourceData-NLP and train the models using partial annotation training (Ding *et al.* 2023).

We used a fine-tuned object detection visual model (YOLOv10) to perform the segmentation of figure images into panel. In the future, this approach could be replaced using large multimodal language models, with dual text and image understanding. Multi-modal models might benefit from the joint information provided by the image of the figure and the text of the caption to process complex figure in a way that comes close to how human view and analyze scientific figures.

While treating the tagging and characterization of biomedical entities as a sequential pipeline shows promising results, it is affected by error propagation. The rise of large language models (LLMs) such as GPT and others has demonstrated their capabilities in various NLP tasks when formulated as text-to-text tasks (Raffel *et al.* 2020). However, their performance in NER tasks has been reported to remain subpar (Jiménez Gutiérrez *et al.* 2022), especially in the biomedical field (Deußer *et al.* 2023). The rapid progress in LLMs suggests, however, that the text-to-text approach with generative models may improve in the future.

Several approaches could be explored to enhance the models’ generalization capabilities, particularly for rare entity types such as subcellular components and diseases, where current performance is lower. Our experimental investigations, which involved entity masking during training to force models to learn purely from contextual cues, did not yield significant improvements in generalization performance. This suggests that more sophisticated approaches may be necessary for addressing the challenges associated with infrequent bioentities.

One promising strategy involves ontology-guided data augmentation, leveraging the hierarchical relationships and synonyms in established resources such as Gene Ontology for subcellular components or Disease Ontology for diseases. Such structured augmentation could systematically expand the representation of rare entities while preserving biological validity and ontological consistency. Recent advances in synthetic data generation using large language models in the biomedical domain (Guo *et al.* 2023, Beeri *et al.* 2024) present another viable approach—employing generative AI to create biologically plausible training examples that enhance the diversity of expressions for underrepresented entity classes, thus mitigating biases.

Furthermore, hybrid methodologies that incorporate partial annotation training approaches (Mayhew *et al.* 2019, Ding *et al.* 2023) would facilitate the integration of specialized datasets focusing on particular entity types, even when these datasets do not conform to the complete annotation schema of SourceData-NLP. Such approaches could effectively supplement the primary dataset with targeted, entity-specific annotations from external sources, thereby addressing current generalization limitations while maintaining annotation quality and biological accuracy.

An exciting potential application of SourceData-NLP is the construction of a large knowledge graph tailored for molecular biology. Knowledge graphs are structured representations of information where entities and their interrelationships are depicted as nodes and edges, respectively. By incorporating the semantic roles obtained from our novel task, we can systematically identify the experimental roles of biological entities in scientific results published in the field of molecular biology literature. This enables the representation of the experimental design in terms of entities linked to the respective “controlled variable” and “measured variable”. Thus, it becomes possible to build a knowledge graph with entities as nodes and directional relationships that represent the causal hypothesis tested in the reported experiments, with various potential applications arising.

First, constructing biological networks with directed edges that represent cause-and-effect relationships can facilitate the discovery of mechanisms and the generation of hypotheses. For example, researchers investigating the effect of a drug on specific tissue could identify potential intermediate mediators by searching for paths connecting these nodes in the causal network topology. Similarly, this approach could facilitate the computational identification of putative regulatory motifs, such as master transcription factors that orchestrate genetic programs; however, rigorous experimental validation is essential to confirm such predictions. The network architecture itself could serve as the basis for organizing literature into a structured index. In preliminary work, we applied unsupervised clustering and automated ontology-building algorithms to relationships extracted from bioRxiv preprints, suggesting that this approach is feasible; however, significant methodological refinements and independent validation studies are necessary to ascertain the quality and utility of this approach.

Curated biological resources often link annotations to the publications that provide the respective supporting experimental evidence. For example, the Gene Ontology annotations in the Uniprot Knowledgebase are usually linked to specific publications. The SourceData dataset and its curation workflow opens the possibility to link annotations at the level of individual experiments providing a more granular and direct reference to underlying evidence, thus reinforcing the integration between knowledge bases, controlled vocabularies and published scientific results. By releasing the SourceData-NLP dataset, we hope to help the development of suitable models and annotation pipelines that will facilitate the annotation of scientific figure and maintaining this annotation at scale.

## Supplementary Material

btaf685_Supplementary_Data

## Data Availability

The data and models underlying this study are available in the following resources: Scripts to generate SourceData-NLP from the raw SourceData annotations: https://doi.org/10.5281/zenodo.17897161 SourceData-NLP in pre-tokenized format: https://doi.org/10.57967/hf/0495 Source code for fine-tuning the NLP models: https://doi.org/.5281/zenodo.17897181 Fine-tuned object detection model: https://doi.org/.10.5281/zenodo.17897195 Fine-tuned PubMedBERT base for NER: https://doi.org/10.57967/hf/7206 Fine-tuned PubMedBERT large for NER: https://doi.org/10.57967/hf/7208 Fine-tuned BioLinkBERT base for NER: https://doi.org/.57967/hf/7207 Fine-tuned BioLinkBERT large for NER: https://doi.org/10.57967/hf/7209 Fine-tuned PubMedBERT base for assignation of experimental roles for gene products: https://doi.org/10.57967/hf/7210 Fine-tuned PubMedBERT large for assignation of experimental roles for gene products: https://doi.org/10.57967/hf/7212 Fine-tuned BioLinkBERT base for assignation of experimental roles for gene products: https://doi.org/10.57967/hf/7211 Fine-tuned BioLinkBERT large for assignation of experimental roles for gene products: https://doi.org/10.57967/hf/7213 Fine-tuned PubMedBERT base for assignation of experimental roles for small molecules: https://doi.org/10.57967/hf/7214 Fine-tuned PubMedBERT large for assignation of experimental roles for small molecules: https://doi.org/10.57967/hf/7216 Fine-tuned BioLinkBERT base for assignation of experimental roles for small molecules: https://doi.org/10.57967/hf/7215 Fine-tuned BioLinkBERT large for assignation of experimental roles for small molecules: https://doi.org/10.57967/hf/7217 Fine-tuned PubMedBERT base for assignation of experimental roles for small molecules and gene products in a single step: https://doi.org/10.57967/hf/7218 Fine-tuned PubMedBERT large for assignation of experimental roles for small molecules and gene products in a single step: https://doi.org/10.57967/hf/7219 Fine-tuned BioLinkBERT base for assignation of experimental roles for small molecules and gene products in a single step: https://doi.org/10.57967/hf/7220 Fine-tuned BioLinkBERT large for assignation of experimental roles for small molecules and gene products in a single step: https://doi.org/10.57967/hf/7221 Fine-tuned YOLOv10 model for panel detection: https://doi.org/10.57967/hf/7222
