# Supplementary material for: Integrating curation into scientific publishing to train AI models
Source: Bioinformatics. 2025 Dec 27;42(1):btaf685. doi: 10.1093/bioinformatics/btaf685 (PMC12836429; doi:10.1093/bioinformatics/btaf685)
Supplement: btaf685_Supplementary_Data [file btaf685_supplementary_data.zip › 08-Jan-2026_093651_supp-info.pdf]

A Online materials and methods

A.1 The SourceData-NLP dataset

The curation workflow takes place on the SourceData platform (Liechti *et al.*, 2017). Curation is carried out by professional curators from Molecular Connections[6]. When curators are uncertain, or in some systematically predefined cases, additional information or identifier validation is requested from the authors (see policies in Supplementary Material B). In the EMBO Press editorial process, authors are requested to provide their source data for figures showing relevant experimental results in their papers. These figures are then sent to the curation team, to be annotated following the guidelines described below and Supplementary Material B[7].

A.1.1 Curation process

We provide an overview of the four major steps in the curation process: splitting figures into panels, tagging entities, linking entities to identifiers, and categorizing the role of entities in the experimental design.

Panel segmentation: In the life sciences, figures often consist of multiple panels that depict results obtained through various methods. To ensure efficient curation and representation of experimental designs, annotations are performed at the level of individual panels. Each panel functions as a cohesive unit of research, typically describing results from a single experimental assay and a defined experimental system. The tagging procedures detailed below apply only to panels presenting experimental data. Schematics, computational simulations, overviews, or workflows are therefore excluded from the entity annotation process. Panels without any entity annotations are nevertheless returned by the SourceData API, complete with their accompanying image coordinates and respective captions**.**

Entity tagging: Specific biomedical terms within a panel legend are tagged and classified into eight mutually exclusive entity classes: small molecules, gene products (genes and proteins), subcellular components, cell types, cell lines, tissues, organisms, and diseases. These classes span a range of scales in biological organization, from small molecules to organisms. Generic references to these classes, such as "proteins," "genes," and "cells," are not annotated. Post-translational modifications, mutations, and other attributes of an entity are also not tagged, but only the base terms. To enable the description of the experimental design, we also tag the experimental assays as an additional class.

Entity linking: Biological entities and experimental assays are normalized and linked to their corresponding identifiers in ontologies. Normalization ensures the unambiguous identification of entities. In cases where a single identifier cannot be assigned definitively, data curators are instructed to assign multiple possible identifiers to the entity. The ontologies used in SourceData-NLP, listed in Supplementary Table 1, are carefully selected to facilitate the annotation process, with preference given to curated entries. General definitions are given below and detailed examples are shown in Supplementary Material B.

Entity roles: The entities from the previous step are further categorized based on the experimental design. SourceData-NLP utilizes “role” to represent causal hypotheses that are experimentally tested. There are six defined experimental roles, as described in Liechti *et al.* (2017):

- *Measured variable* — A measured variable is the component that is measured or observed.
- *Controlled variable* — A controlled variable (also called perturbation, intervention, manipulation, alteration, or independent variable) is a component that is experimentally altered. A controlled variable must be targeted and must be controlled. This implies that the experiment must involve the same experimental system across experimental groups and must include a comparison between several experimental groups to test whether the controlled variable causes an effect on the measured variable.
- *Experimental variable* — When a component is used to compare multiple experimental groups, but it is not possible to infer a cause-and-effect relationship between this component and the measured variables of the experiment, the component is said to be an experimental variable.
- *Biological component* — A biological component is a generic category for any experimentally relevant component that does not fit any of the other defined roles within SourceData. Often it will contain the organism, the cell, or a generic treatment that is present across all conditions.

[6] https://molecularconnections.com/
[7] The guidelines are also maintained at https://sourcedata.embo.org/documentation/

Supplementary Table 1: Ontologies to which the SourceData-NLP tagged entities are normalized.

Entity Type	Primary Resource	Secondary Resource
**Small molecules**	ChEBI[1]	PubChem[12]
**Genes**	NCBI Gene[2]	Rfam[13]
**Proteins**	UniprotKB/Swiss-Prot[3]	
**Subcellular components**	Gene Ontology[4,5]	
**Cell types and cell lines**	Cellosaurus[6]	Cell Ontology[14]
**Tissues & organs**	Uberon[7]	
**Organisms & species**	NCBI Taxonomy[8,9]	
**Diseases**	Disease Ontology[15]	MeSH[16]
**Experimental assays**	BAO[10], OBI[11]	

[1] Hastings *et al.*, 2016, [2] Entrez, 2004, [3] The UniProt Consortium, 2019, [4] Ashburner *et al.*, 2000, [5] Carbon *et al.*, 2021, [6] Bairoch, 2018, [7] Mungall *et al.*, 2012, [8] Schoch *et al.*, 2020, [9] Sayers *et al.*, 2021, [10] Visser *et al.*, 2011, [11] Bandrowski *et al.*, 2016, [12] Kim *et al.*, 2021, [13] Griffiths-Jones *et al.*, 2003, [14] Diehl *et al.*, 2016, [15] Schriml *et al.*, 2012, [16] Lipscomb, 2000

- *Reporter component* — A reporter component is used as a proxy to measure or observe indirectly a measured variable of interest to which it is linked as part of a synthetic or engineered construct.
- *Normalizing component* — A normalizing component is a component that is assayed to provide baseline measurements from each experimental group so that the data can be normalized across groups.

  Each experiment must have at a minimum a measured variable. In cases where important biological entities (e.g. "Measured Variable" or "Controlled Variable") are not explicitly mentioned in the figure legends text, the entities are added as 'floating tags'. Floating tags are, like figure-legend-based entities, linked to the respective identifiers.

Non-entity tagging for experimental assays In addition to entities, SourceData also tags non-entity terms. In particular, the experimental assay used to observe the measured variables of an experiment is tagged. The experimental assays are normalized to identifiers either from the BioAssay Ontology (BAO) or from the Ontology for Biomedical Investigations (OBI). Other non-entity tags are time-related variables and physical variables. A time-dependent variable such as "time course" or "age" is added when the experimental design includes a time-dependency. Physical variables refer to particular physical experimental conditions, e.g., `cold exposure`, `footshock`, etc.

A.1.2 Validation and quality control

The SourceData curation workflow includes a quality control step in which a second annotator performs spot checks on the annotations. This process helps identify and rectify inconsistencies in the labeling of biological entities that are inevitably introduced by human annotators during the workflow. Since the curation process spanned several years, the guidelines were continuously refined to address systematic and common annotation errors.

To address systematic errors outside the workflow, the following corrections were implemented:

1. Exclusion of panels lacking at least one "measured variable" (the entity under study).
2. Verification of entities with different IDs but identical text to ensure that differences were not due to annotation errors.
3. Compilation of a list of overly generic terms (e.g., "image," "percent," or "cell" – see the complete list in Appendix D) that annotators commonly used, and removal of annotations associated with those terms. These terms are listed in Supplementary Material B.4.2.

These measures have been implemented across the entire dataset, initially by curators and subsequently using graph database queries. To keep track of changes, we maintain multiple versions of the dataset: one reflecting the original annotations (including generic terms) as provided by the annotators, and another in which the tags for generic terms have been programmatically removed. Both versions of the dataset are publicly available, and a detailed changelog documenting these revisions is provided in the HuggingFace dataset repository at https://huggingface.co/datasets/EMBO/SourceData. These interventions have improved the accuracy of our annotations, yet the ultimate effectiveness of these corrections depends on the expertise and vigilance of our human annotators in identifying patterns and inconsistencies.

B SourceData guidelines

B.1 Introduction

Experiments in cell and molecular biology involve the manipulation, observation, and description of biological entities. Biological and chemical entities can be entire organisms, a subset of their constituents, or part of the experimental environment.

> **Note:** In this document, the terms entity and component are used interchangeably.

> **Example:** Calcium, oligomycin, p53, mitochondria, liver, mus musculus, synapse, and HeLa cells are entities.

> **Example:** The cell cycle, apoptosis, wound healing, or type II diabetes are not entities.

SourceData description of the data presented in scientific figures specifies the entities that are relevant to the scientific meaning of the data. Annotation of attributes of such entities, biological processes, or diseases is not yet part of the SourceData specification described in this document.

In the following sections of this document, we define the key concepts used in the SourceData annotation process, including the partitioning of composite figures into coherent panels, the tagging of entities, their assignments to types and roles, and their normalization using external identifiers.

B.2 Partitioning figures into panels

Conventional figures are composed of multiple panels and are associated with a description, the figure legend (or figure caption), that explains the content of the figure. While figures tend to present a heterogeneous mixture of experimental designs and assays, individual panels are much more coherent. SourceData annotation is therefore carried out at the level of individual panels.

B.3 Linking panel image and to panel caption

A panel should be defined as a subset of a full figure such that all of the data points/measurements/observations included in the panel are comparable to each other in a scientifically meaningful way. This requires coherent data processing and analysis with an underlying experimental design that enables valid inference about the parameters or relationships of interest. In the majority of cases, panels correspond to the visual panels that authors spontaneously delineate.

Each panel must be associated with its specific panel legend. This association is performed explicitly by trained annotators who match individual visual panels with their corresponding textual descriptions in the figure caption. The inherent structure of scientific figure captions, which typically reference panels through alphabetical or numerical designations, facilitates this process. In cases where figures contain a single panel or lack explicit demarcation, annotators assign the designation 'A' to maintain systematic coverage. This standardized approach ensures that all panels are included in the annotation process, with no content excluded from subsequent entity identification and role classification tasks.

> **Note:** It is often possible to generate a panel legend by including the appropriate textual fragments of the full figure legend. In some instances, multiple non-contiguous fragments need to be spliced together to form a coherent description of a single panel.

B.3.1 Panel Selection for Annotation

The first step in the curation process involves determining which panels are eligible for entity tagging. To be selected for annotation, a panel must report experimental data. Panels that present schematics, computational simulation results, overviews, or workflows are excluded from the annotation process.

This selective approach ensures that the annotations focus on panels containing experimental data that can be meaningfully analyzed for experimental variables, measured outcomes, and biological entities.

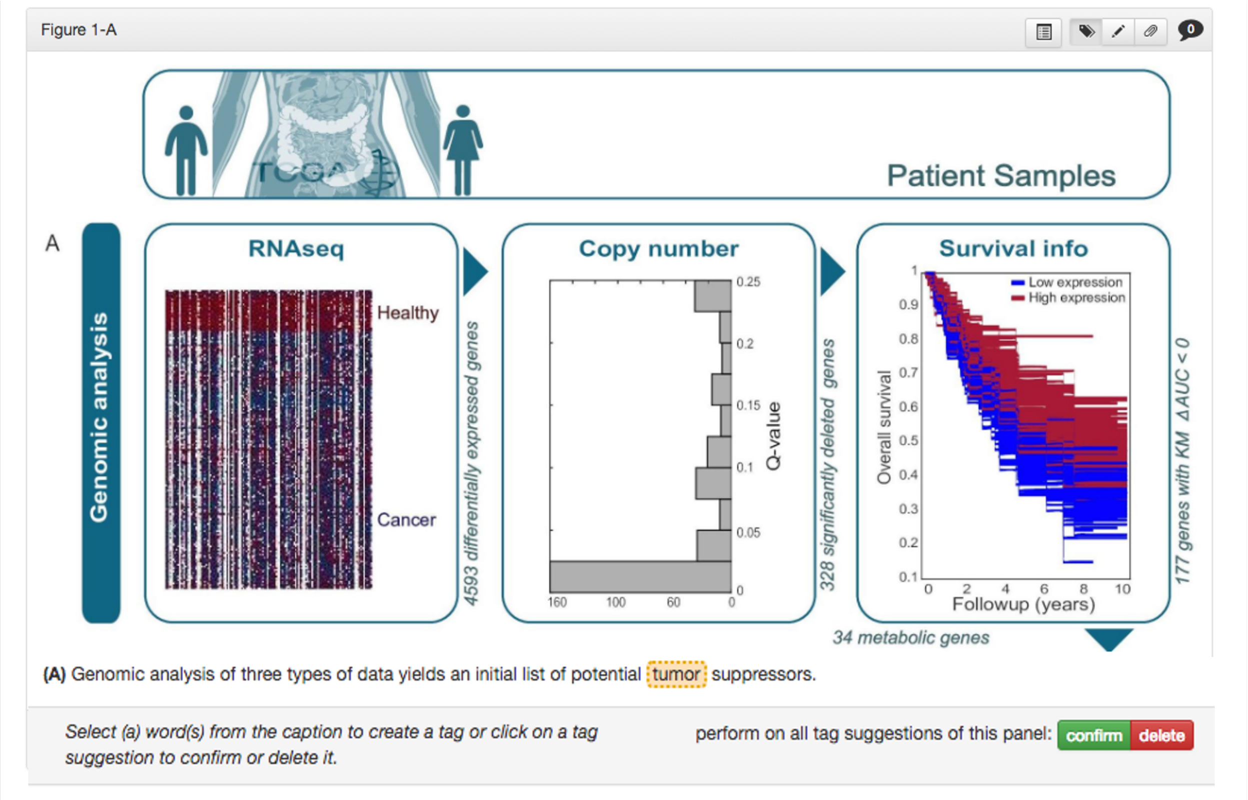

Supplementary Figure 2: Schemes like the one shown above do not need to be annotated.

B.4 Tagging entities

The primary source of information for SourceData annotation is the text of the panel legend and the image of the figure. Relevant terms from the legend or from the image are attached to a tag that specifies their type and role and that can be further linked to identifiers from external biological databases.

B.4.1 Term Tagging in Figure Legends

Within the text of panel legends selected for annotation, terms corresponding to specific biological and chemical entities should be systematically tagged. In general, generic terms referring to broad classes of biological components (e.g., 'proteins', 'cells', 'animals') should not be tagged unless they refer to the object of an assay.

Some terms such as those referring to proteins or genes can be appended with prefixes or suffixes that indicate a post-translational modification, a mutation or other variations of the actual base term. In such cases, pre- or suffixes should be left out and only the base term should be tagged. In other cases, a prefix is added to an entity to denote a species origin, in which case the prefix should be kept.

> **Example:** If the cancer-related mutant form of B-RAF is mentioned as `B-RAF(V600E)` in the text of the legend, the suffix `(V600E)` indicating the mutation should be ignored and only `B-RAF` should be tagged. Similarly, if `p-Akt1` is designating the phosphorylated form of Akt1, only `Akt1` should be tagged and the prefix 'p-' should be left out.

> **Example:** The protein dMyc refers to the Drosophila Myc protein homolog and should be tagged as `dMyc`.

> **Note:** Some components are engineered by assembling or fusing multiple sub-components, which should be tagged individually. For example, the term `RAS-GFP` referring to a fusion protein between GFP and RAS should be annotated with two tags: `RAS` and `GFP`.

Note: In some instances, a fusion construct of multiple entities can be created and referred to within the text via a shorthand symbol generated by authors. In this case, floating tags should be created to refer to the individual components of the fusion construct rather than tagging the shorthand symbol as an entity.

B.4.2 Adding terms missing from the figure legend

Terms can be added as floating tags to complement the description of an experiment with entities that are missing from the text of the legend. These entities typically appear in the image of the figure but not in the legend.

To save time and focus on the most important entities, the use of floating tags should be restricted only to entities with role `controlled variable`, `measured variable`, or `experimental variable`. If one or several of the 3 elements are missing, they should be annotated as floating tags.

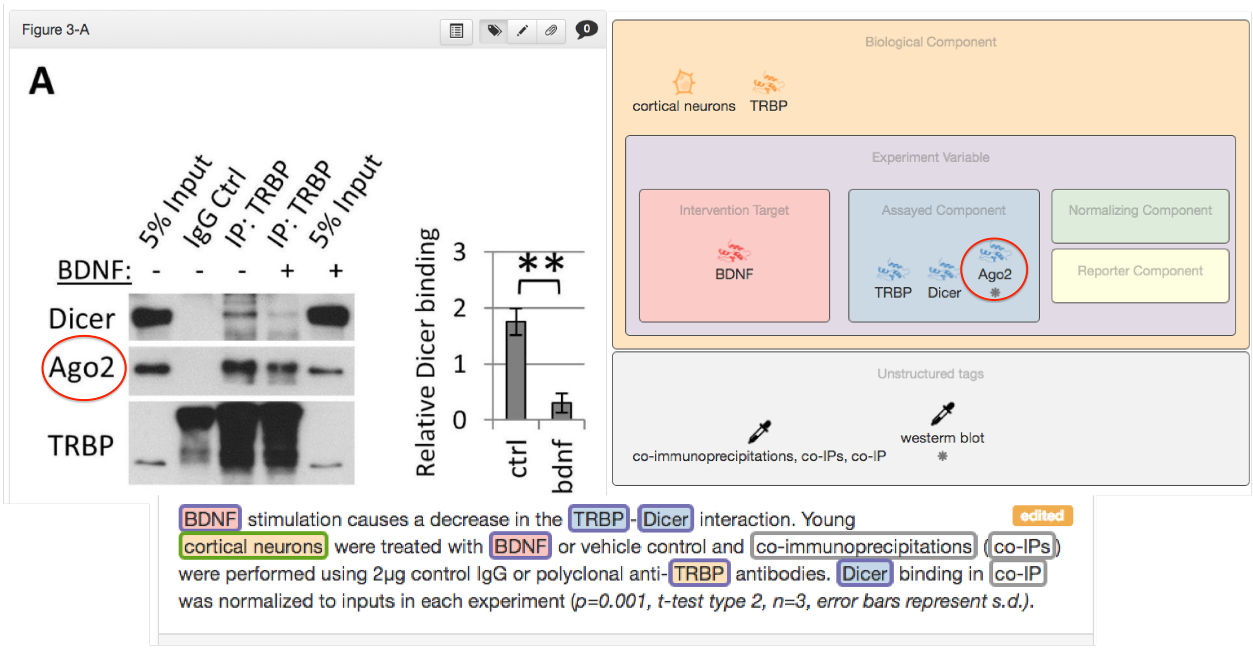

Supplementary Figure 3: Key elements missing in the figure caption should be added as a floating tag: in this example, the measured variable component AGO2 is missing and was added as a floating tag (circled in red). The curation interface marks floating tags with an asterisk (*).

When a generic term is used, such as `cell` or `transcripts`, to refer to a specific entity, i.e., a specific cell line or a specific mRNA, a floating tag should be used to refer explicitly to the specific entity.

B.5 Entity types & normalization

B.5.1 Types

Entities are assigned to one of seven types spanning successive levels of biological organizations (Supplementary Table 1). Each type is mutually exclusive. If an entity is linked to an identifier from an external resource, it should use the resource associated with its type according to Supplementary Table 1.

Example: `ATP` is a small molecule, `creb1` is a gene, `CREB1` is a protein, `the Golgi apparatus` is a cellular component, `HEK293` is a cell line, the `retina` is a tissue, and `Saccharomyces cerevisiae` and `PhiX174` are organisms.

If an entity does not fit any of the predefined types, the undefined type is assigned. By definition, undefined entities cannot be linked to any external resources.

Note: Undefined entities are tagged as such to enable a retrospective analysis of whether additional resources and types should be added in a subsequent version of the SourceData model.

In omics experiments, the number of entities measured is too large to be all listed explicitly. It is then possible to represent the experiments by adding as a floating tag the reserved words `multiple components` and assigning the appropriate type.

B.5.2 Linking to standard identifiers (normalization)

In the normalization process, entities should be linked to one or several identifiers of the external resources corresponding to the entity's type (see Supplementary Table 1). If an entity is linked to multiple identifiers, it must mean that there is uncertainty about the exact identity of the entity.

> **Example:** If the term `Akt` is used to refer to the mouse protein Akt, it is unclear whether it refers to the Akt1, Akt2, or Akt3 isoform. As such, the term will be normalized to the external identifiers `Uniprot:P31750; Uniprot:Q60823; Uniprot:Q9WUA6` corresponding to Akt1, Akt2, and Akt3, respectively.

> **Note:** In the case of entities that are normalized to identifiers from ontologies and taxonomies (subcellular components, cell types, tissues, and organisms), uncertainty about the identity of the entity should be expressed by normalizing it to a sufficiently generic concept in the ontology/taxonomy. For example, the strain `HSV-1 (F)` does not have a specific entry in the NCBI Taxonomy database but can be normalized to the more generic taxon `HSV-1[NCBI Taxonomy:10304].`

Linking reporter components or normalizing components to an external identifier is optional.

Identifiers pointing to curated records of external databases should be preferred over identifiers referring to non-curated records. If relevant records exist both in the primary and secondary resources listed in Supplementary Table 1, identifiers from the primary resource should be used.

B.6 Entity roles

Biological components listed in the caption of a figure each play a different role in the experimental design: some components are altered in a controlled manner, others remain untouched by the experimenter, and some are directly or indirectly measured to perform measurements or observations. Accordingly, the following roles are defined:

- Biological component
- Measured variable
- Controlled variable
- Reporter component
- Normalizing component
- Experimental variable

> **Note:** For all types of entities, if there are multiple instances of the same entity in the figure legend, all instances of the tag should be captured.

B.6.1 Biological components

A biological component is a generic category for any experimentally relevant component that does not fit any of the other defined roles within SourceData. Often it will contain the organism, the cell, or a generic treatment that is present across all conditions.

B.6.2 Measured variables

A measured variable is the component that is measured or observed.

> **Example:** The proteins detected on a Western blot are the measured variables except the loading control, if any, which is considered as a normalizing component (see below).

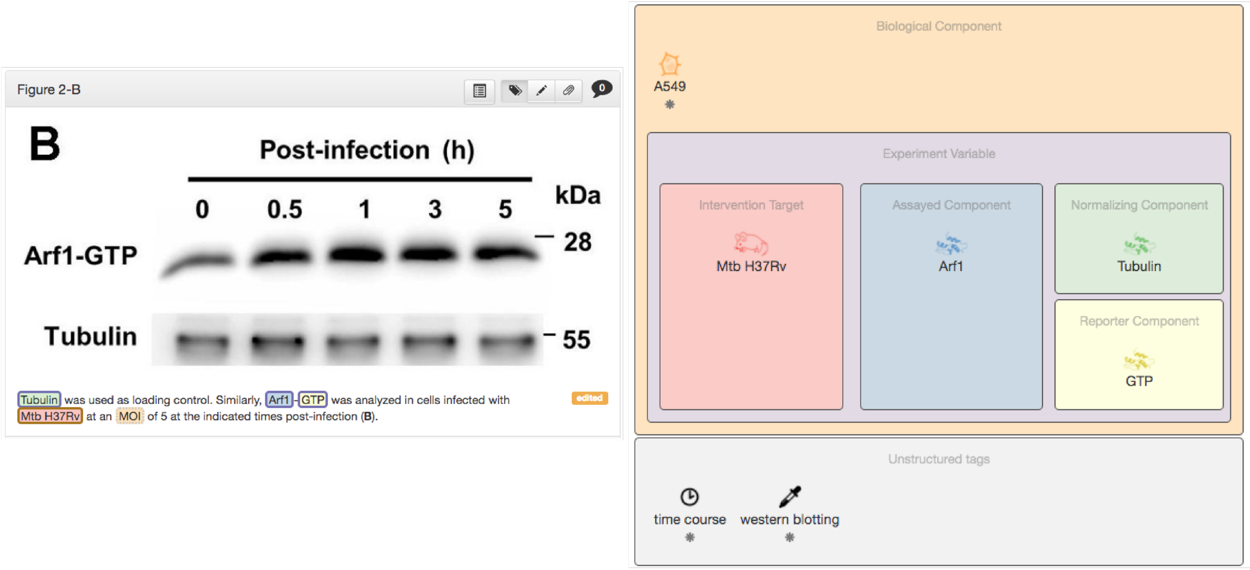

Supplementary Figure 4: In the example above, tubulin is the normalizing component.

Note: If a molecular marker, for example, the protein `EEA1`, is measured to visualize a higher order structure, for example, endosomal vesicles, the marker (EEA1 in this example) is tagged as a measured variable. The higher-order structure (endosomal vesicles in this example) is tagged as a measured variable only if it is explicitly highlighted on the image or a property of the entity (such as number/localization) is mentioned in the text of the legend.

B.6.3 Controlled variables

A controlled variable (also called perturbation, intervention, manipulation, alteration, or independent variable) is a component that is subjected to a targeted experimental intervention. A component is a controlled variable only if the experiment involves the same experimental system across experimental groups and includes a comparison between several experimental groups where the controlled variable is specifically altered in order to test whether it causes an effect on the measured variable.

Example: The function of the gene creb1 can be investigated by comparing creb1 wt (control group) to creb1-/- knockout (test group) mice; in this experiment, creb1 is the controlled variable. If, and only if, it is appropriately controlled, the purpose of such an experiment is to infer a cause-and-effect relationship, whether direct or indirect, between the controlled variable and the measured variable.

Warning: If a drug (cycloheximide, for example) is applied across all experimental groups, it is not considered a controlled variable, since there is no control group to compare the effect of the drug across conditions. A controlled variable must be controlled. Accordingly, in such a context, the drug should be tagged as a biological component. Similarly, if a cell strain harboring the same genetic mutation is used across all experimental groups, the mutated gene is not a controlled variable but a generic biological component.

Note: The target of an experimental manipulation is usually tagged as a controlled variable. Small molecules such as drugs, inhibitors, agonists, and other pharmacological compounds are usually considered as the controlled variable when their effects are compared across experimental groups. An exception is when a small molecule (for example, doxycycline, IPTG, arabinose) is used to manipulate the activity of an engineered circuit controlling the actual entity of interest (for example, a gene whose expression needs to be varied), in which case the entity of interest is considered as the controlled variable and the triggering compound (doxycycline, IPTG, arabinose) is captured as a biological component.

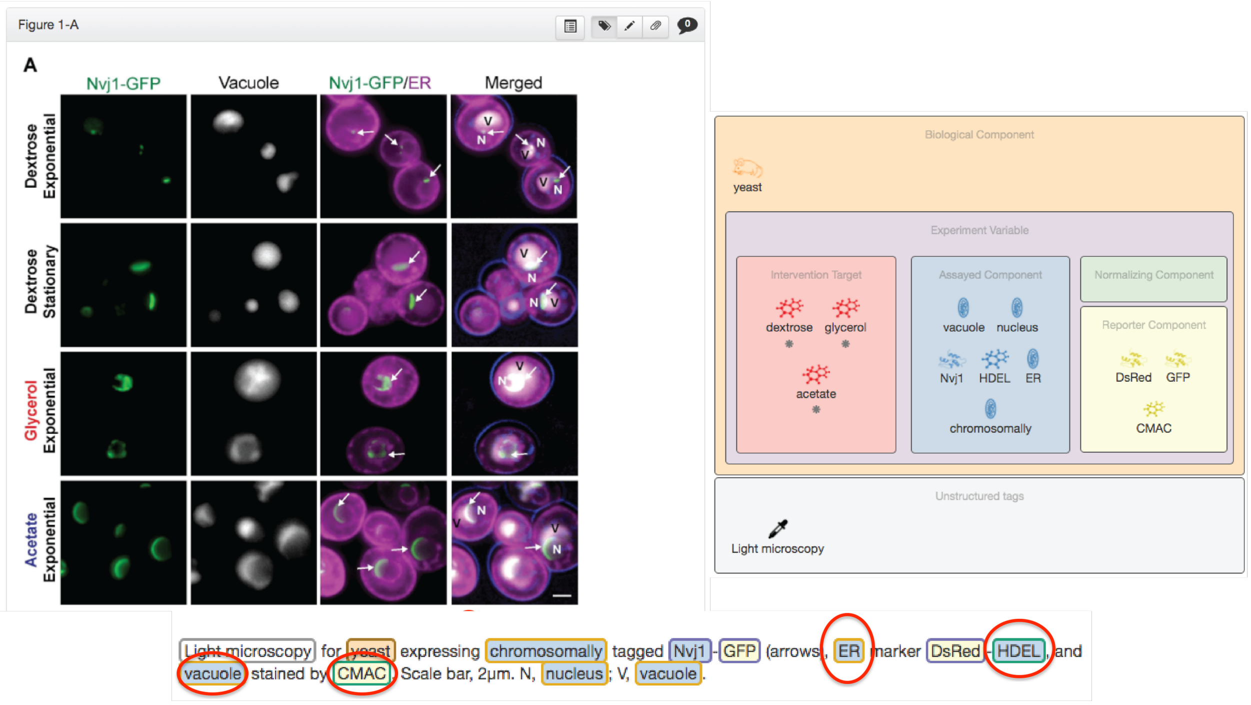

Supplementary Figure 5: In this case, HDEL is an endoplasmic reticulum (ER) marker, so it is captured as a measured variable. In addition, because there is a specific reference to the ER in the legend, ER is also captured as a measured variable. Note that in this example there is a second marker, CMAC, which is, however, a reporter as is therefore captured as a reporter for vacuole, which is labeled as a measured variable.

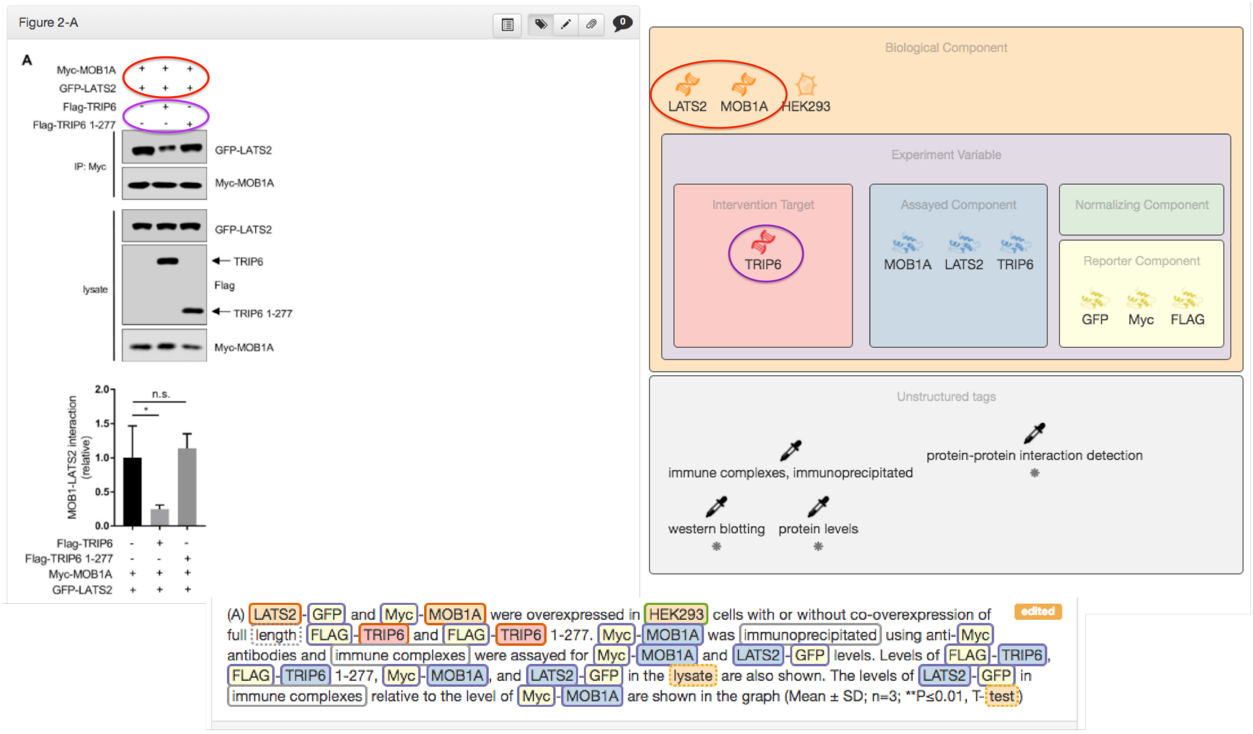

Supplementary Figure 6: MOB1A and LATS2 are both overexpressed across all conditions in this experiment, i.e., they are not controlled for. The only entity that is differentially manipulated in this experiment is TRIP6. Thus, both MOB1A and LATS2 are considered biological components and TRIP6 is considered the controlled variable.

Example: If cells are treated with different doses of the PKA inhibitor `H89`, `H89` is tagged as the controlled variable.

Note: In experiments that test the action of an entity over time, the entity is tagged as a controlled variable only if a control group is tested or if the `time = 0` is also shown as a point of comparison. The time t=0 is essentially equivalent to a control condition.

Example: In a siRNA-mediated knockdown experiment, the gene targeted by the siRNA is tagged as a controlled variable.

Note: A controlled variable *must* involve controlled experimental conditions. It is therefore common that control experimental groups are treated with a neutral compound, for example, the solvent used to dissolve the administered drug. By convention, such components MUST be assigned the generic role of biological components.

In transfection experiments for overexpression, the main entity of the construct used for transfection should be labeled as a controlled variable of type gene and if detected, the protein should be tagged as a measured variable of type protein.

B.6.4 Reporter components

A reporter component is used as a proxy to measure or observe indirectly a measured variable of interest to which it is linked as part of a synthetic or engineered construct.

Example: A RAS-GFP fusion protein includes the RAS protein as a measured variable and GFP as a reporter component.

Example: The luciferase gene can be used as a reporter gene to monitor the transcriptional activity of a given gene promoter, which is the actual measured variable of interest.

Linking reporter components or normalizing components to an external identifier is optional.

B.6.5 Normalizing components

A normalizing component is a component that is assayed to provide baseline measurements from each experimental group so that the data can be normalized across groups.

Example: The proteins beta-actin or GAPDH are often assayed to serve as loading control in Western blots and are then tagged as normalizing components.

Linking normalizing components to an external identifier is optional.

B.6.6 Experimental variables

When a component is used to compare multiple experimental groups but it is not possible to infer a cause-and-effect relationship between this component and the measured variables of the experiment, the component is said to be an experimental variable.

Example: If the expression of a given gene is measured across tissues and cell lines, including liver, muscle, brain, HEK293, and HeLa cells, the tissues or cell types are tagged as experimental variables.

B.7 Tagging experimental assays

In addition to entities, SourceData is also tagging non-entity terms. In particular, the experimental assay used to observe or measure the measured variables of an experiment is tagged and normalized to identifiers either from the BioAssay Ontology (BAO) or from the Ontology for Biomedical Investigations (OBI).

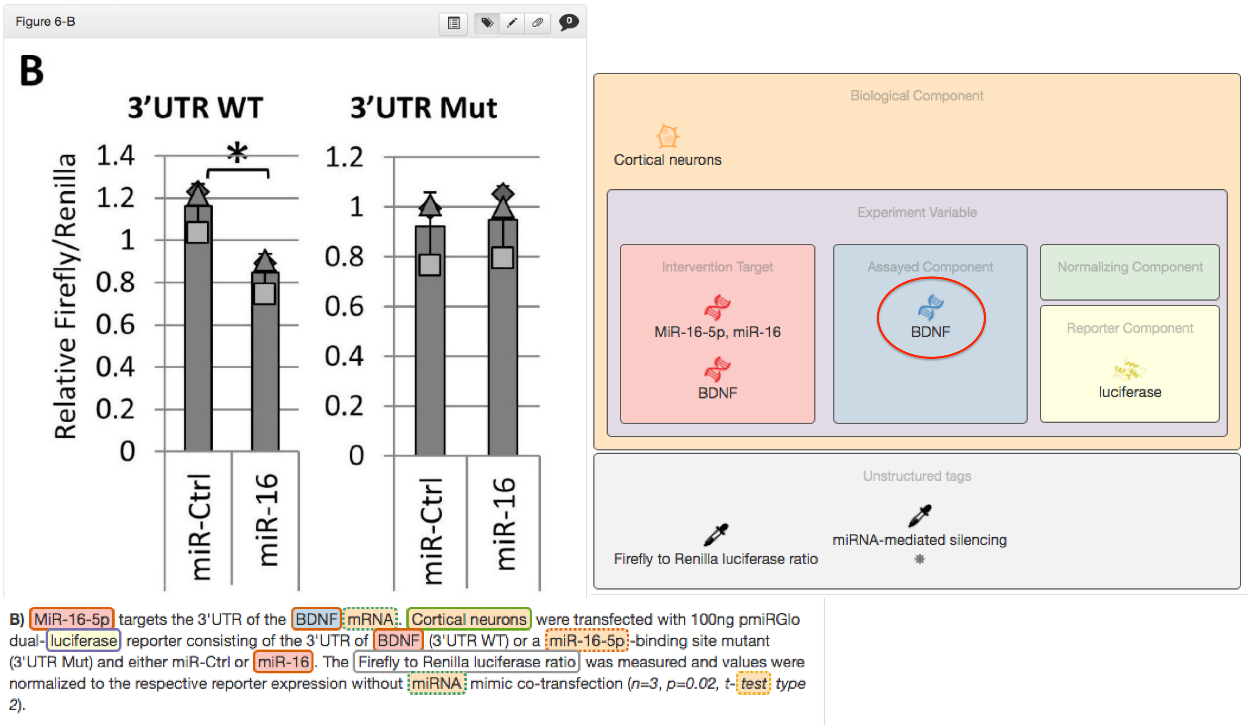

Supplementary Figure 7: In this luciferase experiment, what is being measured is the effect of miR-16-5p on the 3'UTR of Bdnf, so `Bdnf` is labeled as the measured variable.

> **Note:** SourceData only captures experimental assays used to collect data, i.e., associated with the measured variable. The experimental assay used to induce the controlled variable should not be tagged. In addition, SourceData strives to capture the experimental assay itself and not necessarily the METHOD. If both the assay and the method are explicitly mentioned in the figure legend, SourceData captures both. If not, only the experimental assay is captured, either as a tag in the figure legend or as a floating tag if missing.
>
> **Example:** If a figure represents images from an immunostaining, it suffices to annotate `immunostaining` as the experimental assay and there is no need to add a floating tag for `microscopy` if this is not present in the figure legend.

B.8 Time-related variables

Add a floating tag `time course` or `age` to indicate a comparison of a controlled variable or a measured variable over time within an experiment.

B.9 Physical variables

A Physical variable refers to particular experimental conditions, e.g. `cold exposure`, `footshock`, etc. Add them only when explicitly mentioned in the figure legend.

B.10 Special cases: Protein complexes, Fluorescence-Activated Cell Sorting (FACS) experiments, cell cycle phases, and DNA staining

Some experimental designs are unique. For the following cases, these guidelines should be observed:

- Protein complexes: although the intuitive normalization for protein complexes would be of type protein, it is more adequate to assign them the type `subcellular component` because the Gene Ontology (GO) database contains normalized references for protein complexes. An example of this would be RNA Polymerase II, which is made up of a number of individual subunits.

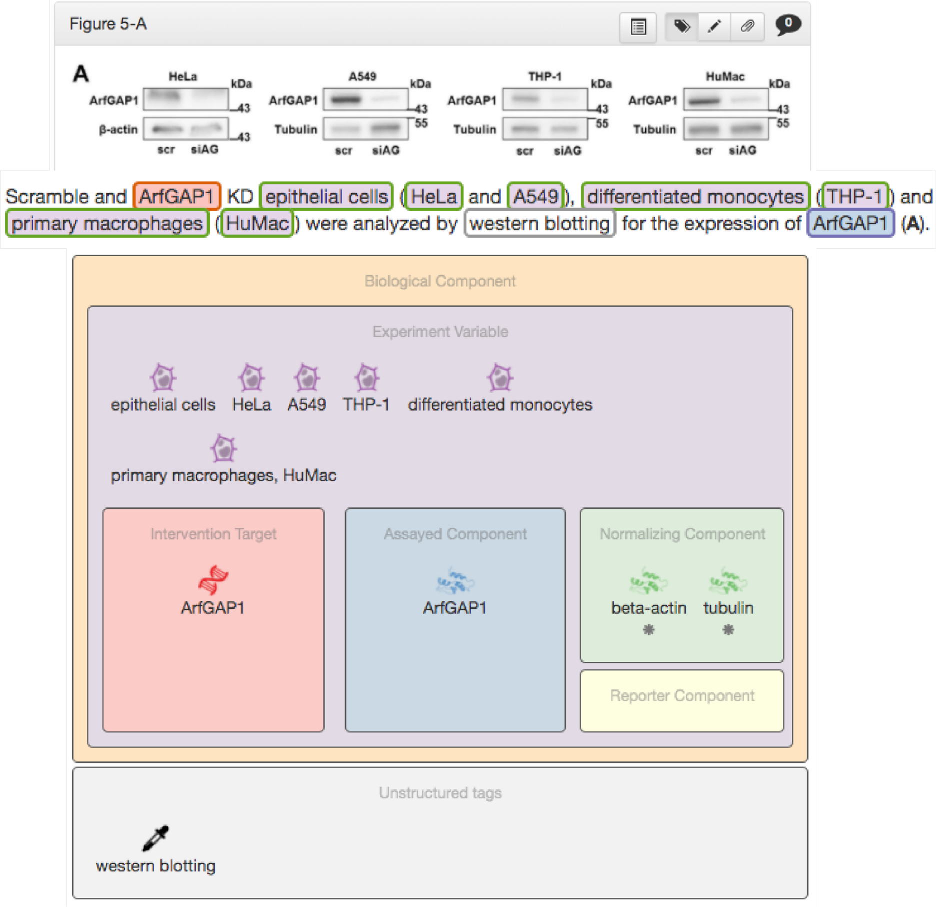

Supplementary Figure 8: In this example, the expression of the `ArfGAP1` protein (the measured variable) is assayed in six cell types upon siRNA silencing of ArfGAP1 gene (the controlled variable). The cell type is not a "measured variable". It also cannot be considered as subject to a targeted intervention. It is therefore assigned the more generic role "experimental variable".

- FACS experiments: for FACS experiments, SourceData captures both the cells and the DNA (or whichever element is stained and sorted) as measured variables.
- Cell cycle phases: at present, SourceData does not include cell cycle phases as time elements. Cell cycle phases may be added when appropriate to experimental variables or biological components as the class `unknown`.
- DNA staining: for DNA stains like BrdU, EdU, etc., SourceData captures DNA as the measured variable and `BrdU staining` as the experimental assay.

B.11 Representing 'omics' experiments

For experiments performing a large number of measurements (>15-20), for example in metabolomics, genomics, transcriptomics, and proteomics, the measured variables cannot be listed individually. The following guidelines are then followed:

- The reserved expression `'multiple components'` should be included as a floating tag, with the relevant entity type, and with the role measured variable.
- Both the measurement type (protein expression, protein-DNA interactions, protein-protein interactions, etc...) and the assay technology (experimental platform such as the sequencing platform, mass spec platform, etc...) as mentioned in Materials & Methods should be tagged as experimental assay, if necessary as floating tag.
- The experimental system that is profiled should be added as a biological component, even if it requires adding a floating tag.

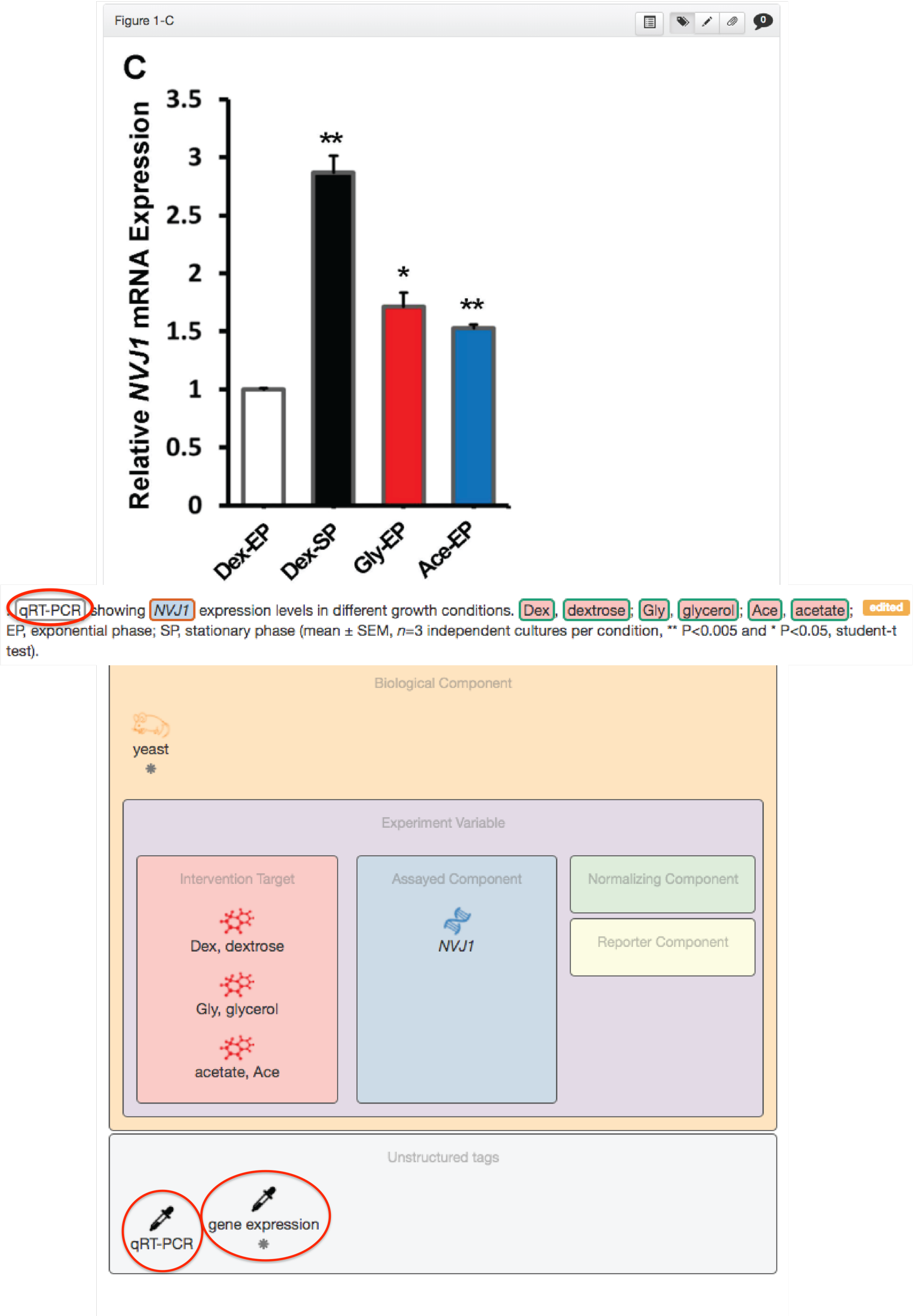

Supplementary Figure 9: In this example, the experimental assay is `gene expression measurement` and the method is qRT-PCR. Because qRT-PCR is mentioned in the figure legend, it is captured. However, `gene expression` is also added as a floating tag to capture the experimental assay for the experiment.

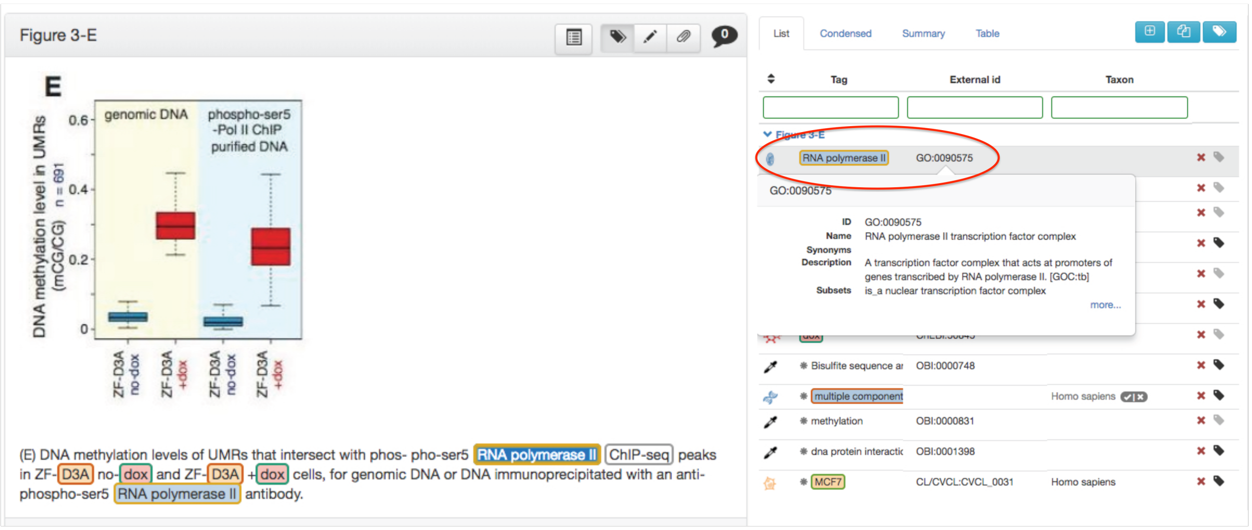

Supplementary Figure 10: Notice how in this example RNA-Pol-II is assigned the type `subcellular component`, which can be normalized to the GO database.

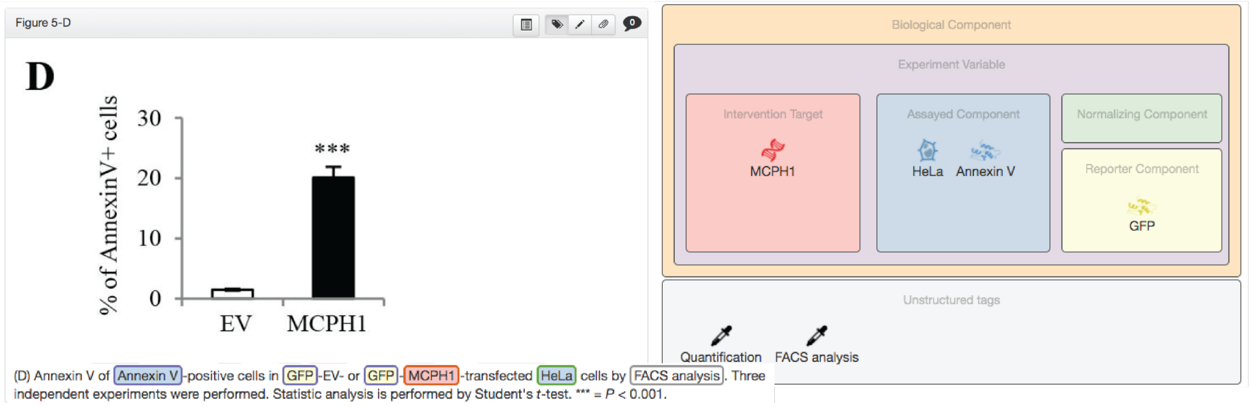

Supplementary Figure 11: In this example, HeLa cells are stained with AnnexinV to measure cell death. Both HeLa cells and AnnexinV are captured as measured variables.

> **Example:** In proteomics, if mass spectrometry is used to measure protein-protein interactions in HeLa cell extracts, for example, the terms `mass spectrometry` (BAO:0000055) and `protein-protein interaction assay` (BAO:0002990) will both be added as experimental assay floating tags, as well as `HeLa` (CL/CVCL:CVCL_0030) as a general biological component that specifies the experimental system.

B.12 Guidelines for author queries

When in doubt about the normalization of an entity, authors can be queried via the validation interface. The following should always be queried to ensure accurate annotation: disease, cell lines, strains, cell types, and molecules if these are normalized to PubChem.

An example of the queries send to authors during the external QC approach described in Section 2.2.2 is shown in the Supplementary Table 16.

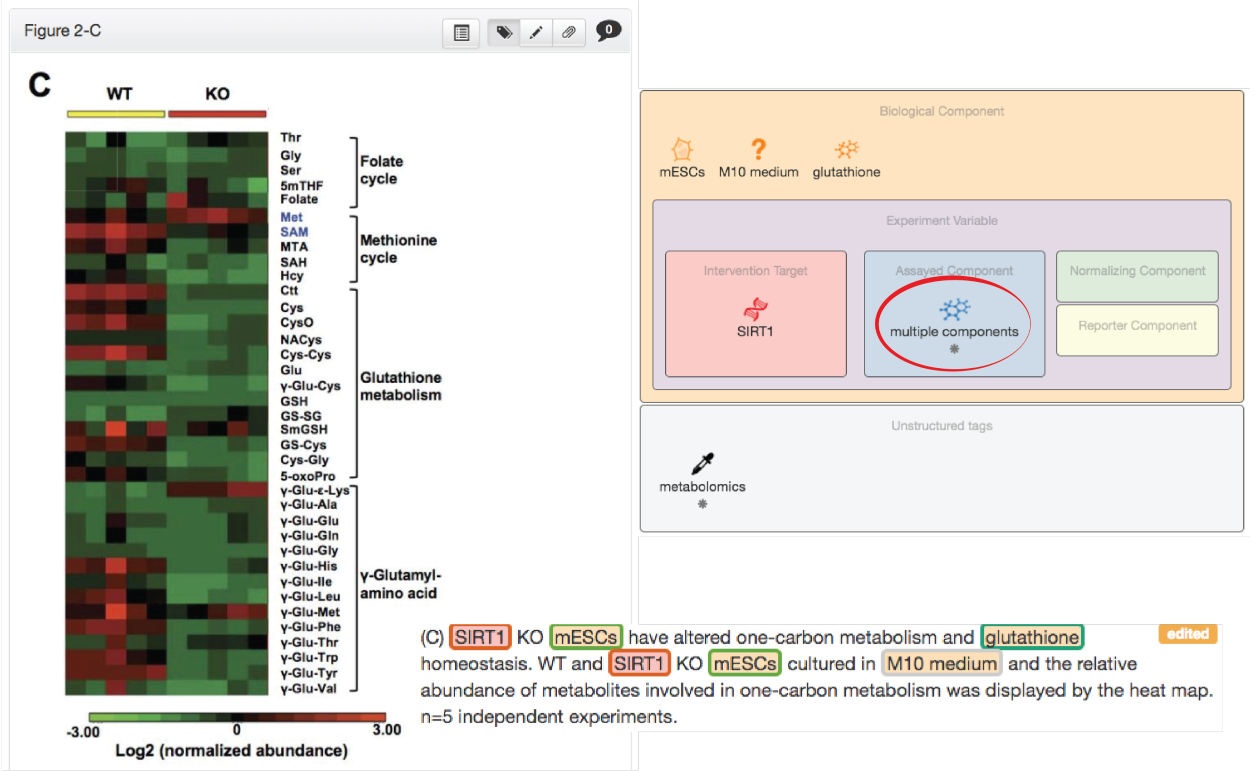

Supplementary Figure 12: Note how individual metabolites are captured under the expression `multiple components` in this example.

C Benchmarking of NLP tasks using SourceData-NLP

To highlight the usefulness of SourceData-NLP, we conducted a set of experiments to train and benchmark the best existing language models across the different tasks supported by our dataset. Previous works have consistently shown that biomedical language models such as PubMedBERT (Gu *et al.*, 2022), and BioLinkBERT (Yasunaga *et al.*, 2022) that use a biomedical vocabulary (i.e., where the tokenization model was trained only on biomedical texts) outperform both biomedical language models that use a general vocabulary (BioBERT Lee *et al.*, 2020; BioMegatron Shin *et al.*, 2020; BioMedRoBERTa Gururangan *et al.*, 2020) and general domain language models (BERT Devlin *et al.*, 2019; RoBERTa Liu *et al.*, 2019). Accordingly, the experiments of this work were carried out with the PubMedBERT and BioLinkBERT models using both their large and base versions.

We perform the benchmarking on four tasks: splitting the caption into panels ("panel segmentation"), NER, and the novel semantic interpretation of experimental roles. The best-performing model for each task is available on the EMBO repository hosted on the HuggingFace transformers Hub[8]. We next describe the fine-tuning procedures in detail.

C.1 Panel segmentation, NER, and semantic interpretation of experimental roles

All the tasks are formulated as token classification problems. In this configuration, the language models are fine-tuned to assign labels to individual tokens. Labels are assigned based on the IOB schema (Ramshaw and Marcus, 1995), which denotes the (B)eginning tokens of entities, tokens (I)nside entities, and tokens (O)ut of entities.

For the panel segmentation task, the entire figure caption is used as input for the model. Tokens are labeled as `O` or `B-PANEL_START` to indicate the starting position of each panel. The results of the panelization benchmark are shown in the Supplementary material E.1.

The tasks of NER and semantic interpretation of experimental roles utilize the text of distinct panels as input. Subsequently, tokens are categorized into one of eight distinct and non-overlapping classes. These include `O`, signifying no biological entity of interest; `B-GENEPROD` and `I-GENEPROD`, representing gene products; and additional classifica-

[8]https://huggingface.co/EMBO

Supplementary Table 12: Hyper-parameters used for fine-tuning.

Parameter search space	Fixed fine-tune param.
train batch size	32 (8)
eval batch size	64
epochs	2
gradient accumulation steps	1 (4)
learning rate	1e-4
lr scheduler	cosine
adam β_1	0.9
adam β_2	0.999
adam ϵ	1e-10
weight_decay	0.0
adafactor	True

Note: The numbers in parenthesis show different values for large (>300M parameters) models.

tions for other biological entities like subcellular components, cell types, cell lines, tissues, organisms, diseases, and experimental assays, as annotated in SourceData-NLP.

The task of assigning experimental roles to the entities is specifically applied to gene products as they are by far the most prevalent entities with these roles. We approach this task in three different ways, as explained in the main text. First, we make the semantic classification task purely dependent on context, masking the gene product entities with the special `[MASK]` token and assigning the labels `CONTROLLED_VAR`, `MEASURED_VAR`, or `O` to them ("context-only approach"). Secondly, we mark the position of entities with a special token without masking them ("marked-entity approach"). In this case, the models can learn both from context and from the identity of the entities. Finally, we train the models to recognize the roles of gene products without any extra information, forcing the models to perform both, NER and role interpretation on a single step ("single-step approach").

To ensure reproducibility, we maintained the same fine-tuning hyperparameters for all tasks as summarized in the Supplementary Table 12. Model parameters were optimized with AdamW with the cross-entropy loss. To avoid memory issues the large version of the models were trained using a batch size of 8 compared to 32 for the base model. To achieve comparable results between both model sizes, we used the same learning rate and 4 gradient accumulation steps for training large models. The reported results are the average F1 scores obtained from 5 consecutive runs of the experiments using different random seeds.

Training is carried out on an NVIDIA DGX Station with 4 Tesla V100 GPUs for 2 epochs. Each fine-tuning takes about 20 minutes for a base model and about an hour for a large model, which has approximately 3 times more parameters.

C.2 Multimodal segmentation of compound figures

C.2.1 Model Training and Evaluation

The YOLOv10 object detection model was fine-tuned using distributed training across 4 Tesla V100 GPUs for 30 epochs. We employed both default and optimized hyperparameters, with training images standardized to 720 pixels and batch size dynamically adjusted for optimal performance. The training pipeline utilized 16 data loading workers to ensure efficient data processing and throughput.

Model evaluation incorporated several optimized parameters. We reduced the inference image size to 420 pixels to balance speed and accuracy, while setting the confidence and IoU thresholds to 0.3 and 0.9 respectively. These thresholds were empirically determined to optimize the trade-off between detection sensitivity and prediction quality. Additional optimization included limiting maximum detections to 20 panels per image and implementing half-precision inference for computational efficiency. To evaluate our panel segmentation approach, we created a dataset of 13,039 figures with annotated panel boundaries, split into training (11,735), validation (651), and test (653) sets. The metrics used to measure the model performance was mAP_{50} (mean average precision with 50% intersection-over-union threshold) and mAP_{50-95} (average precision across IoU thresholds from 50% to 95% in 5% increments). With this configuration, the YOLOv10-based object detection algorithm achieves accuracies of $mAP_{50} = 98.2\%$ and a $mAP_{50-95} = 87.0\%$. The test set contains 653 images with a total of 3,067 panels and reporting a total of 52 false negative errors.

C.2.2 Error Analysis

Analysis of the 52 false negatives revealed systematic patterns in the detection pipeline. The primary failure mode (14 cases) involved composite panels containing multiple subpanels. In these cases, the model correctly identified individual subpanels but generated excessive detections by treating each subpanel as independent (Supplementary Figure 13). These cases represent an inherent ambiguity in panel annotation rather than a model deficiency.

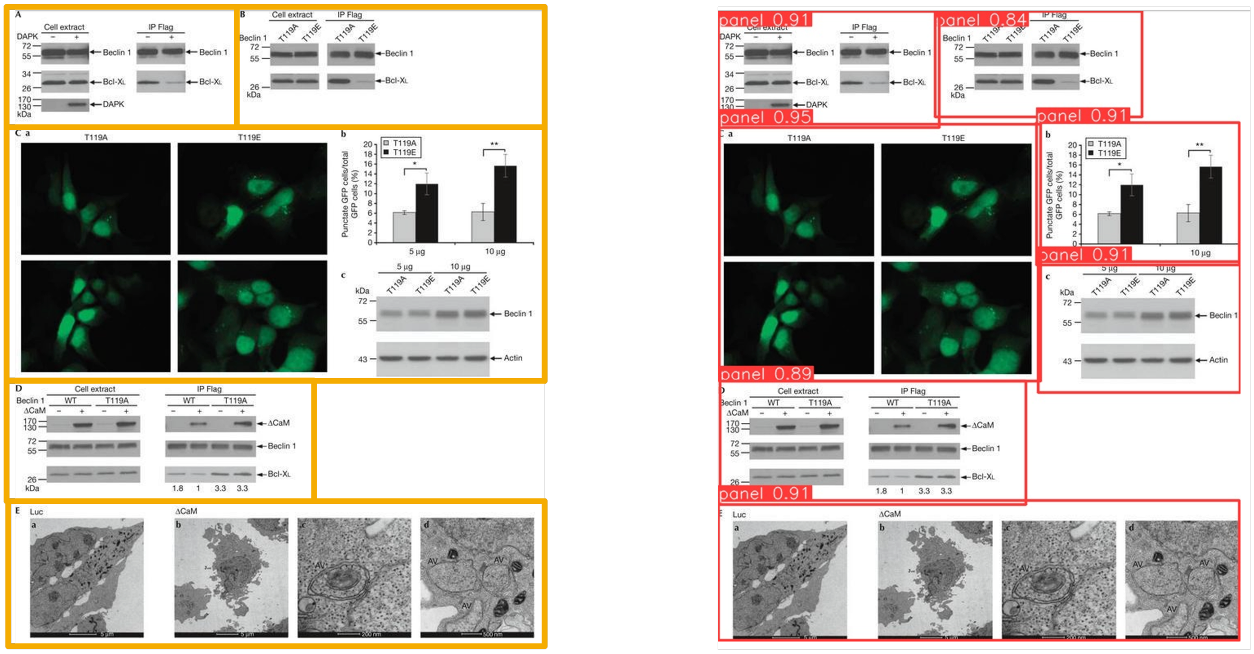

Supplementary Figure 13: Example of a figure with a panel composed of multiple subpanels. Ground truth is shown in the left with yellow boxes and the object detection inference is shown in the right with red boxes and the detection confidence of the model. The panel C is label as a single panel, but is composed of the subpanels Ca, Cb, and Cc, that have been correctly identified by the model.

Panel overlap constituted the second most frequent error (9 cases), occurring in densely-packed figures where space optimization led to partial panel overlaps. While adjusting the IoU parameter could potentially address these cases, such modifications risked compromising overall model performance (Supplementary Figure 14).

Western blot panels account for 6 undetected cases, mainly in image corners (Supplementary Figure 15). The distinctive vertical format and lane organization of these experimental readouts may represent an underrepresented pattern in the training data. This systematic weakness suggests the need for targeted data augmentation or specialized model components for this common experimental format. Despite western blots being well-represented in our training dataset, this error pattern suggests a possible challenge with the spatial context recognition rather than simple class imbalance.

Less frequent but notable error modes included panels with non-rectangular boundaries (2 cases) and interconnected panels with linking schematics (2 cases). These cases highlight the inherent limitations of rectangular bounding box approaches for complex scientific visualizations. Additionally, we observed decreased detection accuracy for panels positioned at figure bottoms and those deviating from conventional grid layouts, suggesting potential biases in the model's spatial understanding.

C.2.3 Panel-caption-matching

The panel-caption matching pipeline leveraged OpenAI's GPT-4o multimodal model through API calls configured with temperature=0.5, balancing contextual understanding with output consistency. The system prompt was designed to extract panel-specific captions while preserving both generic and specific descriptive elements, with outputs structured as JSON objects for automated evaluation.

Combining the two steps we obtain a figure segmentation and panel matching accuracy of 97.7%, that translates into a single panel miss every six papers, assuming an average of 20 panels per paper, as is extracted from our SourceData-NLP dataset.

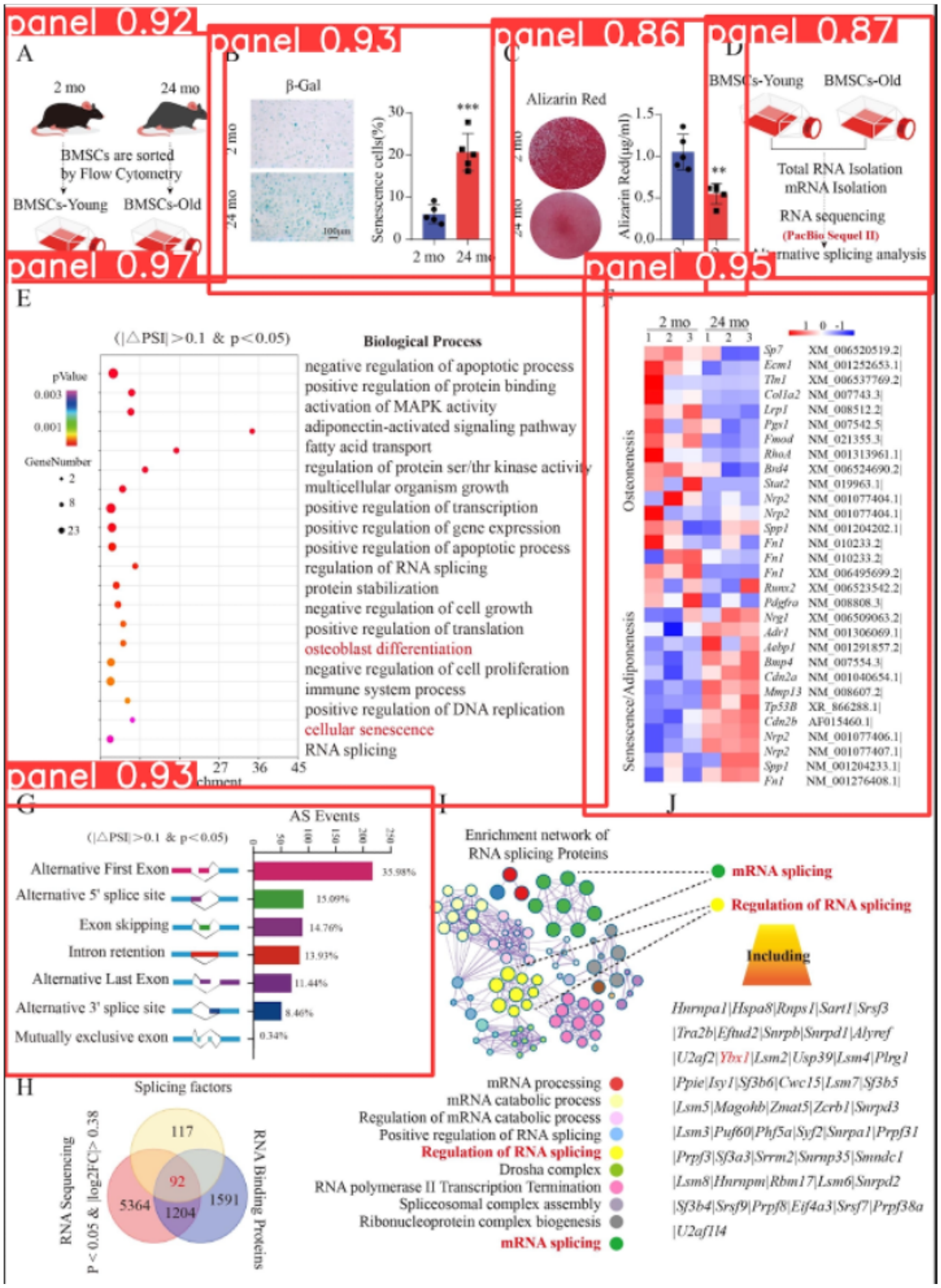

Supplementary Figure 14: Example of a figure schematics linking several panels. Another of the systematic sources of panel misidentifications by the object detection model. The panels H, I, and J are not identified by the model.

We make the source code used for this task openly available for researchers to help accelerate research and reproduce our results. It can be found at `https://github.com/source-data/soda_image_segmentation`. The repository includes detailed documentation, configuration files, and evaluation scripts to ensure reproducibility.

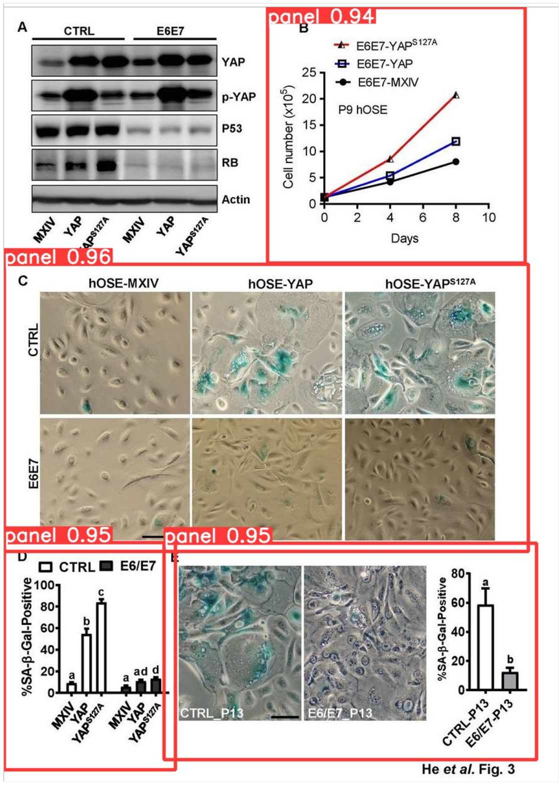

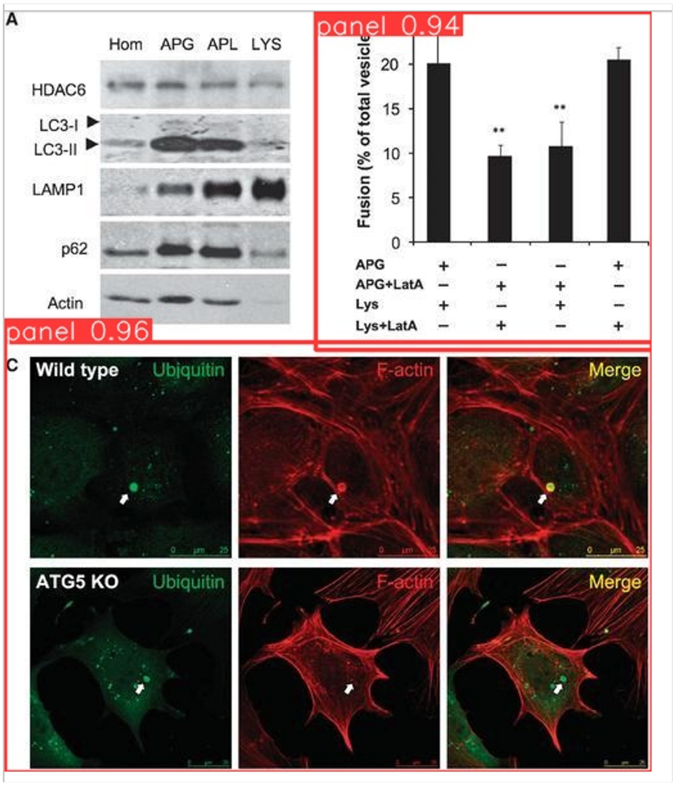

Supplementary Figure 15: Example of figures with not identified western blot panels in the corner of the figure. The panel A is not identified by the model.

System prompt:

```
"""You will receive a text with the caption of a scientific figure. This
    figure will be generally composed of several panels. Extract the
    relevant part of the figure caption so that it matches the panel given
    as an image file. If a generic description of several panels is in
    place, return the generic and the specific descriptions for a given
    panel. Make sure that the information in the panel caption you return
    is enough to interpret the panel. For simplicity in post-processing
    begin the caption always with 'Panel X:' where X is the label of the
    panel in the figure.

Output format:
```
{
    "panel_label": "X",
    "panel_caption": "Description of the panel."
}
```
"""
```

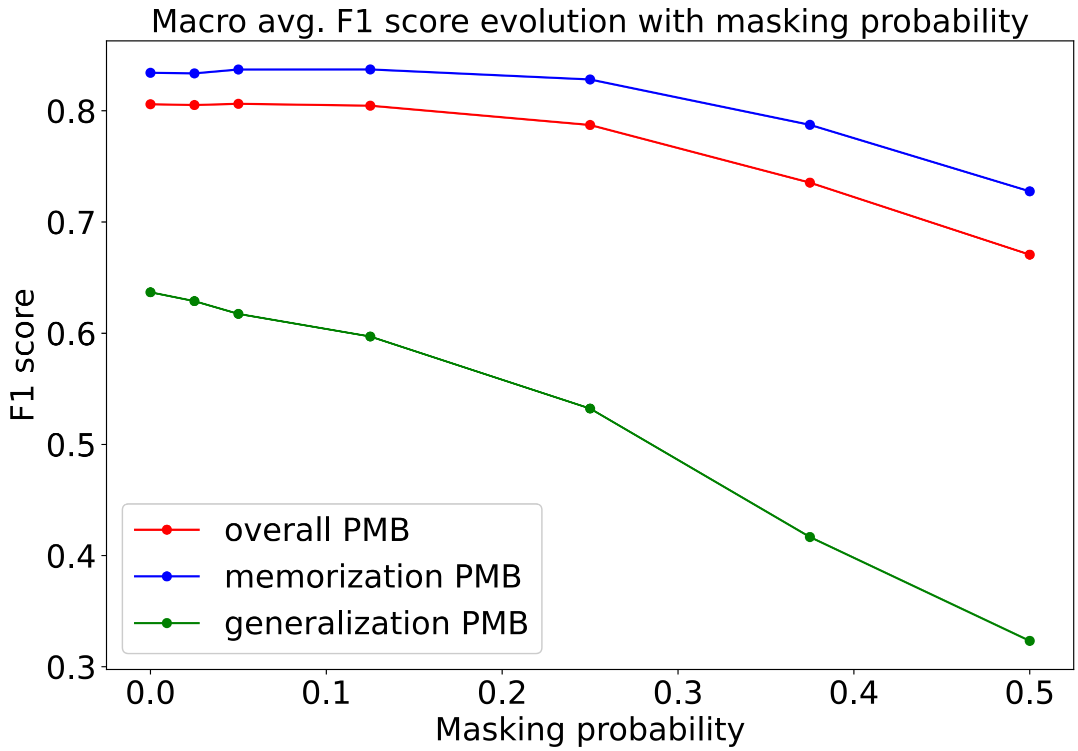

Supplementary Figure 16: The performance in terms of general (depicted in red), memorization (in blue), and generalization (in green) is demonstrated through the F1 score of PubMedBERT base, measured against the masking probability.

D Removal of generic terms from the annotations

During our examination of the assembled dataset, we noticed that numerous general terms —such as age, DNA, cells, and animals— had been tagged. We chose to omit a subset of these non-specific terms from the dataset. This decision was motivated because these terms are often too vague or encompassing. As such, they are not ideal for generating precise descriptions of experimental setups. These are explicitly marked to guide future annotation efforts by the dataset curators. Additionally, we have created a correction patch that eliminates all tags corresponding to these identified terms, thereby ensuring dataset consistency. A comprehensive, alphabetically ordered list of such terms is shown below. These changes will be available in the version 2.0.2 of SourceData-NLP.

List of omitted non-specific terms: age, aggregates, all, amino acids, analysis, and, animal, animals, antibiotics, area, assay, bacteria, bacterial, based, body weight, cell death, cell, cells, cellular, chromatin, cleaved, complex, concentration, contralateral, core, count, counts, cytoplasmic, cytosolic, dark, distance, DNA, duct, ductal, ducts, embryo, embryos, female, females, fetal, fetus, fibers, fluorescence intensity, fluorescence, fluorescent, gas, gland, glands, hand, heatmap, IHC, images, images, individual, intensity, ipsilateral, laser, length, level, levels, light, line, littermates, lung tumor, male, males, membrane, micrograph, micrographs, morphology, muscle, muscles, nuclear, number, numbers, parasite, parasites, patient, patients, percent, percentage, percentages phenotype, phenotypes, photograph, photographs, plant, plants, primary cell, primary cells, protein, proteins, pulse, puncta, punctae, quantification, ratio, rest, RNA, SEM, sequence, size, stained, staining, stem, strain, tailed, TEM, temperature, test, tests, the, tumor, tumors, virus, viruses, weeks, weight, white.

Supplementary Table 16: Results of the panel segmentation task for overall, memorized tokens and non-memorized (generalization) tokens. The best-performing model for each case is shown in bold.

Pretrained model	Size	F1 overall	F1 memo.	F1 gen.
PubMedBERT	base	90.5	90.1	94.3
PubMedBERT	large	91.5	91.2	95.2
BioLinkBERT	base	90.8	89.3	95.7
BioLinkBERT	large	**92.4**	**92.0**	**96.5**

E Additional Results

E.1 Panel segmentation task

The panel segmentation task splits figure captions into their constituent panels by assigning a `B-PANEL_START` label to the first token of each panel. We fine-tuned BioLinkBERT and PubMedBERT to evaluate how well these models would perform. Both the models perform similarly as shown in Table 16. We also studied how well they could memorize versus generalize for this task. Panel separators in the biomedical literature are not very varied and tend to be consistent (e.g. A, a), (A), (a)), so most examples were considered "memorized." "Generalized" examples were figures without explicit panel separators, where any word might indicate a new panel. This typically happens with content from publishers who do not include the panel separators in their XML content but only as style CSS classes on the live website. Some high-order panel separators (e.g. Z, O) also required generalization.

The models' overall F1 scores were similar for memorized and generalized examples, as expected given that memorized examples made up over 95% of the data. However, for generalized examples, F1 scores were 4 points higher on average. These encouraging results show that these language models can distinguish panels based on context alone.

Supplementary Table 16: Author Query Templates

ID	Query Type	Query Template
2	Cell line - hierarchy	Kindly, help us assign the correct identifier to this cell line. We have linked the cell line below to the indicated reference ID in cellosaurus but as you know, there are many variations to a given cell line. Please confirm if the the link we have selected is an accurate description of your cell line or if it would be more accurate to link it to a different “daughter cell lines” (under ‘Hierarchy’).
4	Molecule - confirm	Kindly confirm whether the identifier below correctly reflects the molecule you used in this experiment? We believe this to be the most accurate identifier, but please let us know if a more accurate one is available. Thank you very much.
5	Molecule - choose best id	Please help us choose the most accurate identifier for this entity: We found several candidate IDs for the molecule below. Kindly confirm if assigning all identifiers is accurate. Thank you very much.
6	Molecule - No external id	In an attempt to be as exhaustive as possible with our curation, we try to unequivocally identify all the molecules/-chemicals that were of importance in your experiments. We were unable to find an external identifier for this molecule in ChEBI (https://www.ebi.ac.uk/chebi/) or PubChem (https://pubchem.ncbi.nlm.nih.gov/). If you have a reference or order number, we might be able to use that as a guide to find the correct identifier. If you find an accurate identifier in ChEBI, PubChem, or another external database, we would also appreciate if you could provide us with that. Thank you very much.

Continued on next page

Supplementary Table 16: Author Query Templates

ID	Query Type	Query Template
8	Protein - No external id	In an attempt to be as exhaustive as possible with our curation, we try to unequivocally identify all the proteins that were of importance in your experiments. We could not find an external identifier for the protein below in UniProt. Please help us find the most accurate identifier for this entity. If applicable, a reference number from a vendor would also be helpful. Thank you very much.
9	Protein - Species unclear	In an attempt to be as exhaustive as possible with our curation, we try to unequivocally identify all the biological entities that were of importance in your experiments. We were uncertain about the concrete species of origin of the protein and therefore could not assign an UniProt ID. Kindly help us find the most accurate identifier for this entity. A species of origin and/or reference number from a vendor if appropriate would also be helpful. Thank you very much.
11	Protein - Several identif	In an attempt to be as accurate as possible with the curation of your experiments, we aim to correctly assign Uniprot identifiers to all proteins in your experiment. For the protein highlighted below, we found several external identifiers i n UniProt. Kindly review the links below and let us know if assigning all of these identifiers is correct and accurate based on the assay you used and to the best of your judgement. Thank you very much.
13	Gene - No id found	In an attempt to be as exhaustive as possible with our curation, we try to unequivocally identify all the genes that were of importance in your experiments. We could not find an external identifier for the gene below in NCBI gene. Please help us find the most accurate identifier for this entity. Thank you very much.
14	Gene - Multiple identifie	Kindly let us know whether it is accurate to link the listed NCBI gene identifiers to the gene highlighted below. Thank you very much.
15	Gene - Species unclear	Kindly help us to identify the genes used in your constructs in this experiment. Please confirm the species of origin of the construct (e.g. mouse, human) you used for transfection. Thank you very much.
16	Disease - choose best id	Since diseases have many nuances, we want to make sure that we capture the best and most accurate possible annotation for your disease models. We found the identifier below for this disease term but there seem to be more specific children terms. Kindly help us choose the identifier that best describes your model. Thank you very much.
17	Disease - no id found	We were unable to find a good identifier for this disease term, could you please browse these databases and let us know if you find an accurate descriptor of your model? https://www.ncbi.nlm.nih.gov/mesh/ http://disease-ontology.org Thank you very much.
18	Disease - general confirm	Since diseases have many nuances, we want to make sure that we capture the best and most accurate possible annotation for your disease models. We found the identifier below for this disease term but would be thankful if you review and confirm that this is an accurate descriptor of the disease model you used. Thank you very much.
19	Cell types - no id	We were unable to find a suitable id to identify these cells in your experiment. Kindly help us find an accurate identifier in this database: https://www.ebi.ac.uk/ols/ontologies/cl Thank you very much.

Continued on next page

Supplementary Table 16: Author Query Templates

ID	Query Type	Query Template
20	Cell types - general conf	Kindly confirm whether the linked identifier below correctly and accurately describes the cells you describe/use in this experiment? Thank you very much.
24	Protein - general confirm	Kindly confirm whether the mapping we chose in the linked identifier below is accurate for this protein? Thank you very much
25	internal - normalization	[action needed] - mapping or queri is missing. Please add.
26	Gene - general confirmati	Could you please review the linked identifier below and confirm whether this is the best identifier for the biological entity in NCBI gene? Thank you very much
27	Cell line - no ID	We were unable to find a suitable id to identify these cells in your experiment. Kindly help us finding an accurate identifier in this database: https://web.expasy.org/cellosaurus/ Thank you very much.
28	Gene - multiple id's	Please review the linked identifiers below and confirm whether it is accurate to map the gene to all of these variants? Thank you very much
29	Cell type - no id	We were unable to find a goog mapping in NCBI taxonomy for this particular strain. Could you please browse this URL and let us know what the best possible identifier would be? https://www.ncbi.nlm.nih.gov/taxonomy Thank you very much!
30	Virus Strain unclear	We were unable to find a suitable id to identify this virus strain in your experiment. Could you please help us find an accurate identifier in this database? https://www.ncbi.nlm.nih.gov/Taxonomy/Browser/wwwtax.cgi Thank you very much.
31	Gene-species-specific	Kindly help us correctly identify the constructs you used for transfection in this experiment. In this experiment flag-tagged Wdr37 was ectopically expressed in 293T cells. Can you please clarify the species of origin of the Wdr37 gene (e.g. mouse, human) you used for generating the expression vector? Thank you very much.
32	Stably expressing line sp	Could you please help us correctly identify the constructs you used for transfection in this experiment? In this experiment QC25 was expressed in this marker line. Can you please clarify the species of origin of the QC25 gene (e.g. arabidpsis, mouse, human) you used for this line? Thank you very much.
33	Stable TG line	Could you please help us correctly identify the constructs you used for transfection in this experiment? In this experiment WOX5 was expressed in this marker line. Can you please clarify the species of origin of the WOX5 gene (e.g. arabidopsis, mouse, human) you used for this line? Thank you very much.
34	virus strain no ID	In an attempt to be as exhaustive as possible with our curation, we try to unequivocally identify all the biological entities that were of importance in your experiments. We were unable to find an external identifier for this viral strain in the NCBI taxonomy browser. (https://www.ncbi.nlm.nih.gov/taxonomy/) Can you please help us find the most accurate identifier for this virus? Thank you very much.
35	[internal] annotation sel	[Annotation selection] Dear annotators, in this paper please only annotate the following panels: token Thank you!

References

Ashburner, M. *et al.* (2000). Gene ontology: tool for the unification of biology. *Nature Genetics*, **25**, 25–29.

Bairoch, A. (2018). The cellosaurus, a cell-line knowledge resource. *Journal of biomolecular techniques : JBT*, **29 2**, 25–38.

Bandrowski, A. *et al.* (2016). The ontology for biomedical investigations. *PLOS ONE*, **11**(4), 1–19.

Carbon, S. *et al.* (2021). The gene ontology resource: enriching a gold mine. *Nucleic Acids Research*, **49**, D325 – D334.

Devlin, J. *et al.* (2019). Bert: Pre-training of deep bidirectional transformers for language understanding. In *fuck*, pages 4171–4186, Minneapolis, Minnesota. Association for Computational Linguistics.

Diehl, A. D. *et al.* (2016). The cell ontology 2016: enhanced content, modularization, and ontology interoperability. *Journal of Biomedical Semantics*, **7**.

Entrez (2004). *NCBI Gene*. National Library of Medicine (US).

Griffiths-Jones, S. *et al.* (2003). Rfam: an rna family database. *Nucleic acids research*, **31 1**, 439–41.

Gu, Y. *et al.* (2022). Domain-specific language model pretraining for biomedical natural language processing. *ACM Transactions on Computing for Healthcare*, **3**(1), 1–23.

Gururangan, S. *et al.* (2020). Don't stop pretraining: Adapt language models to domains and tasks. In *Association for Computational Linguistics (ACL)*.

Hastings, J. *et al.* (2016). ChEBI in 2016: Improved services and an expanding collection of metabolites. *Nucleic Acids Research*, **44**(D1), D1214–D1219.

Kim, S. *et al.* (2021). Pubchem in 2021: new data content and improved web interfaces. *Nucleic Acids Research*, **49**, D1388 – D1395.

Lee, J. *et al.* (2020). BioBERT: a pre-trained biomedical language representation model for biomedical text mining. *Bioinformatics*.

Liechti, R. *et al.* (2017). Sourcedata - a semantic platform for curating and searching figures. *Nature Methods*, **14**, 1021–1022.

Lipscomb, C. E. (2000). Medical subject headings (mesh). *Bulletin of the Medical Library Association*, **88 3**, 265–6.

Liu, Y. *et al.* (2019). Roberta: A robustly optimized BERT pretraining approach. *arXiv*, **arxiv:1907.11692**.

Mungall, C. J. *et al.* (2012). Uberon, an integrative multi-species anatomy ontology. *Genome Biology*, **13**, R5 – R5.

Ramshaw, L. A. and Marcus, M. P. (1995). Text chunking using transformation-based learning. In *Proceedings of the Third Workshop on Very Large Corpora*, Cambridge, USA. Association for Computational Linguistics.

Sayers, E. W. *et al.* (2021). Genbank. *Nucleic Acids Research*, **42**, D32 – D37.

Schoch, C. L. *et al.* (2020). Ncbi taxonomy: a comprehensive update on curation, resources and tools. *Database : the journal of biological databases and curation*, **2020**, baaa062.

Schriml, L. M. *et al.* (2012). Disease ontology: a backbone for disease semantic integration. *Nucleic Acids Research*, **40**, D940 – D946.

Shin, H.-C. *et al.* (2020). Biomegatron: Larger biomedical domain language model. In *Proceedings of the 2020 Conference on Empirical Methods in Natural Language Processing (EMNLP)*, pages 4700–4706, Stroudsburg, USA. Association for Computational Linguistics.

The UniProt Consortium (2019). Uniprot: a worldwide hub of protein knowledge. *Nucleic Acids Research*, **47**, D506 – D515.

Visser, U. *et al.* (2011). Bioassay ontology (bao): a semantic description of bioassays and high-throughput screening results. *BMC Bioinformatics*, **12**, 257 – 257.

Yasunaga, M. *et al.* (2022). Linkbert: Pretraining language models with document links. In *Proceedings of the 60th Annual Meeting of the Association for Computational Linguistics*, Dublin, Ireland. Association for Computational Linguistics (ACL).
